# Experiences with rehabilitation and impact on community participation among adults with physical disability in Colombia: perspectives from stakeholders using a community based research approach

**DOI:** 10.1186/s12939-019-0923-4

**Published:** 2019-06-03

**Authors:** María Luisa Toro-Hernández, Alejandra Mondragón-Barrera, Sara Múnera-Orozco, Laura Villa-Torres, Wendy Camelo-Castillo

**Affiliations:** 1 School of Physical Therapy, CES University, Medellin, Colombia; 2 Department of Social Medicine, UNC School of Medicine, University of North Carolina at Chapel Hill, Chapel Hill, USA; 3 Department of Pharmaceutical Health Services Research, School of Pharmacy, University of Maryland Baltimore, Baltimore, USA

**Keywords:** Physical disability, Comprehensive rehabilitation, Community participation

## Abstract

**Background:**

Despite representing 70 million people in Latin America, access to comprehensive rehabilitation and participation in the community remains a challenge for persons with disability (PWDs) in the region. Through enactment of the Disability Law, Colombia has made improvements to recognize and address some of the barriers for PWDs, including access to comprehensive rehabilitation. However access remains limited with significant disconnect between perspectives of various stakeholders and the needs of the population. We examined the unique perceptions on access to comprehensive rehabilitation services and participation of PWDs. We also explored the perspective of caregivers of PWDs, rehabilitation professionals, and other stakeholders on the experiences of PWDs. Our goal was to identify gaps in the implementation of comprehensive rehabilitation programs, and barriers to access resources for comprehensive rehabilitation or services that would impact participation of PWDs.

**Methods:**

Qualitative study conducted in 2017. Data was collected from a purposive sample of adults with physical disability, aged 18–44 years, who had received rehabilitation services at a local partner organization and with different backgrounds and experiences with disability. Purposive sampling was also conducted with caregivers, rehabilitation professionals, and other stakeholders. Socio-demographic information was collected and semi-structured interviews were conducted by a research team member, recorded, transcribed and analyzed using a thematic analysis method to identify main themes related to our aim. CES University ethical review board approved this study.

**Results:**

Thirty-two participants were interviewed: eight were male, 42.1 ± 11.1 years old, and 44% (*n* = 14) were PWDs. Three main themes were identified among all the participants: the meaning of rehabilitation, challenges to access services, and participation. Challenges to access services had three sub-themes: barriers to personal mobility, perceptions and knowledge on disability, and navigating the system.

**Conclusion:**

The main focus of rehabilitation as perceived by stakeholders is still on functional rehabilitation. If healthcare personnel is better trained on disability and if those with disabilities are actively involved in the developing these programs, the focus may evolve to a comprehensive and equitable rehabilitation process that fosters full participation.

**Electronic supplementary material:**

The online version of this article (10.1186/s12939-019-0923-4) contains supplementary material, which is available to authorized users.

## Introduction

Seventy million people in Latin America live with a disability, and higher rates are observed among the poorest and most vulnerable [1]. The Economic Commission for Latin America and the Caribbean estimates that 12.6% of the population in the region has a disability which is expected to increase with aging, non-communicable diseases, poverty, armed conflict, and urban and gender violence as contributing factors [2, 3]. Despite the ratification of the United Nations Convention on the Rights of Persons with Disabilities (CRPD) by all countries in Latin America, access to habilitation, rehabilitation and health services by people with disability (PWDs) remains a challenge, limiting opportunities for participation [4, 5]. Access to services is a complex concept that involves availability, physical accessibility, adequate supply, affordability, and acceptability [6]. Therefore, equitable access to habilitation, rehabilitation, and health services depends on a complex interaction between factors at the personal, community, contextual, and system levels [7]. Because access to these services is critical to meet the needs of PWDs and increase participation [8], it is currently being proposed by investigators in the rehabilitation field as an indicator of equity [9, 10].

The International Classification of Functioning, Disability, and Health (ICF) defines disability as an evolving concept that results from the complex interaction between a person with an impairment and personal and contextual factors, with the potential of hindering their participation in society under equal conditions than others [11]. Participation is a human right, which includes involvement in all aspects of life such as civil, political, economic, social, and cultural spheres [4, 11]. Rehabilitation (article 26, CRPD) is a strategy to facilitate participation as it aims to improve health, restore, and maintain long-term functioning [4, 12, 13]. A comprehensive rehabilitation approach includes articulation among services and programs in health, employment, education, and social sectors [4]. The Global Disability Action Plan recognizes the lack of research evidence on the real needs of PWDs as a barrier to the implementation of effective rehabilitation services [14]. In Latin America, rehabilitation research is scarce [15], where limited evidence is available to support policy and decision-makers. This leads to the development of policies and implementation of programs that may not address the real needs of the community [3]. Specifically in Colombia, disability-related research is still at its infancy with minimal knowledge translation [16] and low participation of PWD [14]. Within the Latin American region, there is a need to generate high quality and contextually appropriate evidence to inform program design, implementation, and policy-making [16].

In recent decades, Colombia has had significant advances developing its constitutional and legal framework to protect the human rights of PWD. Specifically, the constitutional reform of 1991 along with the signature (2009) and ratification (2011) of the CRPD, resulting in the enacted Disability Law in 2013 and led to the development of the National Disability Public Policy Plan [17] [18]. However, implementation of this legal framework is still in early stages. Progress reported to the United Nations after the ratification of the CRPD mainly focused on changes in the legal framework [19–21]. Advances in the implementation of programs (e.g. inclusive education and employment opportunities) were few individual success cases in main cities [19–21]. Both the government and the civil society recognized that major challenges persist to achieve an inclusive and rights-based approach systematic change [19–21]. As part of the response to the reports, the United Nations urged the Colombian State to guarantee universal accessibility to all and focus on the needs at the territorial level [22]. There is a need to identify local gaps to effectively advocate for the actions that may be needed [19].

In Colombia, there is a lack of reliable and up-to-date data on disability [19, 20]. Based on the WHO estimates, disability would affect at least 15% of the country’s population, which equals about 7.5 millions of Colombians [3]. The 2005 national census reported a disability-prevalence of 6,3% [23]; while in June 2018 the National Disability Registry (registration is voluntary) reports a 2.8% prevalence [24]. The Registry collects data on socio-demographic characteristic of the PWD, type of activity limitations, access to rehabilitation, among others. Data from June, 2018 show that 50% of young adults and adults have a physical or mobility impairment [24]. Twenty-two percent state that they need rehabilitation (defined as therapies and medicines), where 59% of them do not attend any rehabilitation programs [24]. Only 12% work, 2% have finished an undergraduate program, and more than 80% have an income lower than the minimal wage or no income at all [24]. These numbers reflect some of the profound inequities that PWDs face, despite the existing disability policy framework [24]. Prior to the Disability law, Moreno-Angarita et al. explored the concept and practice of rehabilitation across stakeholders such as program managers, PWD, and academics [25]. Although this study highlighted discrepancies on the understanding of rehabilitation by the stakeholders, with significant focus still on the medical model of disability, it represents the perspectives of stakeholders in Bogota, the country’s capital [25]. There is limited knowledge about the challenges of PWD outside of the capital city. To achieve equity, it is imperative to identify gaps and develop evidence within the regions in the country.

In alignment with the Colombian Disability Research Agenda [26] with our work we aim to generate evidence on the experiences related to access to rehabilitation in a geographical region that has not been previously explored, and with the second largest population with disability in the country [24]. Our approach is informed by previous evidence on the broad challenges experienced by young adults and adults with physical disability. With this lens we examine the unique perceptions on access to comprehensive rehabilitation services and participation of PWDs. We also explored the perspective of caregivers of PWDs, rehabilitation professionals, and other stakeholders on the experiences of PWDs. Our goal is to identify gaps in the implementation of rehabilitation programs, and barriers to access resources for comprehensive rehabilitation or services that would impact participation of PWDs.

## Methods

A qualitative study design was undertaken to explore the experiences related to access and use of comprehensive rehabilitation services and their impact on participation of PWDs in Envigado, Colombia. This work was developed in partnership with a community organization ALFIME. ALFIME offers physical therapy, physical activity, psychology, legal advice, independent living program to PWD, and educational talks offered to users with disabilities and their families. The programs are financed with public funds from the city of Envigado and offered to users based on a sliding scale fee. Many users receive the services at no cost. In consequence, ALFIME is one of the main resources available for PWDs in the city regardless of socioeconomic status.

### Sample

This study was conducted in the city of Envigado. a city of 240 thousand inhabitants in the metropolitan area of Medellin, the second largest city in Colombia. Envigado has lower development than Medellin and Bogota [27] and 40,5% PWD in the city have been reported as excluded from the human capital (defined as deprived from education and health rights) [28]. Through purposive sampling we identified key participants that represented diverse groups in terms of gender, age, and other background characteristics. A basic socio-demographic questionnaire (Table 1) was used to capture these characteristics and to inform the sampling. In Colombia, the socio-economic status has 6 levels (1–6). For the purpose of this study, these were grouped as low (1 and 2), middle (3 and 4), and high (5 and 6). PWDs were eligible to participate if they were ages 18–44 and lived in Envigado. PWD in this age range were selected because in Colombia they represent the second largest group with disabilities after older adults with the lowest access to education and high unemployment rate during their most productive years [24].Physical disability was defined as a permanent physical or mobility impairment affecting their body, upper or lower limbs, dexterity or coordination [24]. In addition, we had a purposive sample of caregivers, rehabilitation professionals, and other stakeholders that reflected different experiences of providing care for PWDs or were involved in diverse initiatives relevant to the population of interest. For the purpose of this study, rehabilitation professionals were defined as those involved in services and programs aimed at improving functioning in PWD. The WHO that recognizes that rehabilitation is cross-sectorial and may be carried out by health professionals in conjunction with specialists in education, employment, social welfare as well as community workers in contexts with limited resources [29].

**Table 1:** Sociodemographic variables included in the questionnaire by participant type

People with disabilities	Caregivers	Rehabilitation Professionals	Other stakeholders
Age	Age	Age	Age
Gender	Gender	Gender	Gender
Health condition that caused the physical impairment Assistive technology used Civil status, Socioeconomic status of the household (low, medium, high) Highest level of education attained Receiving disability pension Occupation	Hours providing care Highest level of education attained by the caregiver Socioeconomic status of the household Occupation of the caregiver	Years working with PWD Occupation area (e.g. health, sports, counseling, etc.)	Type of organization they work for (non-governmental organization, government, academia, private company, independent contractor)

### Procedures

PWDs and caregivers were recruited through referrals by providers affiliated with ALFIME and other local organizations. Rehabilitation professionals were invited to participate from the pool of professionals affiliated with ALFIME, representing various disciplines and perspectives. Other stakeholders were identified through local government agencies, civil organizations, academia, and community leaders.

In-person semi-structured interviews were conducted in Spanish by one of the research team members [MLTH]. An interview guide, with open-ended non-directive questions, was developed for each group of participants and each explored the areas of independence and autonomy, perceptions on disability, perceptions and available resources in rehabilitation, and citizenship. Members of the research team [MLTH, LVT, WCC] developed the guides with insights from experts in ALFIME [see Additional file 1]. The procedures described in this manuscript are part of a larger project that actively engaged the community further through an asset mapping exercise and a community forum. The interviews took place at a private room in ALFIME, or at a location of their preference. Interviews lasted a maximum of 2 h and 27 min and were digitally recorded. Field notes were completed following each interview. Interviews were transcribed verbatim, and data was managed and analyzed using Dedoose Version 8.0.35, web application (2018). Prior to the interview, each participant completed a brief socio-demographic questionnaire (Table 1).

We used thematic content analysis to analyse the data. Techniques used in the analysis included analytical summaries, open coding, identification of thematic codes, and codebook development. Each interview was coded by two members of the team; disagreements were discussed and resolved through involvement of a third team member. Initial analysis allowed identification of major challenges regarding access and use of rehabilitation services. Through subsequent analyses, themes were refined, focused on exploring domains such as the meaning of rehabilitation, challenges to accessing services, and community participation. We generated matrices to compare and contrast patterns across the perspectives of PWDs, caregivers, rehabilitation professionals, and other stakeholders. We assessed data saturation through an iterative analytical process that included conducting the interviews, reviewing field notes, reading and coding the data, and developing matrices. Even though our sample size was limited by financial restrictions, our saturation assessment alongside with data triangulation across participants made us confident that the key themes were saturated.

The trustworthiness aspects of the study are based in the criteria proposed by Nowel et al. [30]. First, credibility is established as MLTH attended ALFIME on a periodic basis which resulted in prolonged engagement, persistent observation, and building rapport with the community. At least one undergraduate student assisted in the interviews and interview debriefs between MLTH and the student were conducted immediately. A weekly update meeting was conducted between MLTH, MAM, and WCC. The thematic content procedure was rigorous as previously described. Second, the transferability is established by sufficient description of the context of the study and its methods Third, the research team continuously audited the process (e.g. analytical summaries, recordings of the raw data, field notes, and transcripts) and a reflexive journal was kept. Therefore, the dependability was achieved. Last, as the study achieved the credibility, transferability, and dependability aspects, confirmability is established [30].

## Results

A total of 35 people were invited to participate. Three men in the PWDs group were not eligible due to age or because they did not have the means to meet for the interview. We interviewed 8 participants per group of interest, for a total of 32 participants. Table 2 presents the socio-demographics information of the 4 groups. For participants in the PWDs group, health condition related to the disability included spinal cord injury, neurodegenerative conditions, congenital conditions, and rheumatoid arthritis, among others. All PWDs used at least one assistive technology device (e.g. manual/power wheelchair, walker, etc.) and half were single. Four caregivers provided care between 1 and 3 h per day, two between 4 and 6 h, and 2 more than 7 h daily. The rehabilitation professionals had on average 17 years of experience in the disability field, 4 provided support to PWDs through legal counseling, individual or family counseling and 4 were involved with school, sports or program administration. Among other stakeholders, 2 were involved with academia, 3 with the local government, and the remaining were consultants or independent contractors.

**Table 2:** Socio-demographic characterists of the participants

Characteristic/Participant		Users with disability	Caregivers	Rehab. Professionals*	Other stakeholders*
Age (median (IQR))		32(5,5)	51(12,5)	52(4,5)	36(5)
Gender	Female	6	6	7	5
	Male	2	2	1	3
Education level	Complete high school or less	2	4	Information not collected	
	Associates degree or more	6	4		
Socioeconomic level	Low	2	1	Information not collected	
	Middle	5	6		
	High	1	1		
Occupation		2 students 4 unemployed or w/o occupation 2 retired/pension	4 caregivers/ house wives 2 unemployed or w/o occupation 1 retired/pension 1 employed	8 employed	8 employed

*IQR: inter quartile range; w/o: without; *Two rehabilitation professionals and 4 among other stakeholders are persons with disability

Three main themes emerged from the interviews: the meaning of rehabilitation, challenges to access services, and community participation. Table 3 provides a summary of the themes by type of participant.

**Table 3:** Main themes and sub-themes related to access to comprehensive rehabilitation and summary of experiences, by type of participant

Themes	Sub-Themes	PWDs	Caregivers	Rehabilitation Providers	Other stakeholders
Meaning of rehabilitation		Inclusive of physical and psychological therapy, sports, and education	Includes therapies and assistive technology	Strategy to promote independence, both in the PWD and their caregivers	A process that “frees” the PWD and their family
		It is necessary to overcome fear of leaving the house and improve quality of life	Necessary so PWD can do things without help and reduce their care burden	Should be tailored to individual needs with a multidisciplinary/multilevel approach	Beyond health and includes sports, recreation, education, employment, peer mentoring, and services for caregivers
		Value of rehabilitation seen through peers who have had a successful outcome	Rehabilitation as a tool for acceptance	A path to independence, to be able to decide on your own	Requires articulation between PWD, their families, and providers
Challenges to access comprehensive rehabilitation services	Barriers for personal mobility	Homes of PWDs are inaccessible		PWDs lack ability to navigate accessibility barriers with assistive technology	PWDs lack ability to navigate physical barriers with assistive technology
		Built-environment barriers in public places	Public places with lots of stairs, no ramps or elevators	Built-environment barriers in public places	Accessibility challenges in public spaces
		Lack of accessible, reliable, and affordable public transportation	Lack of accessible and affordable public transportation	Lack of accessible, reliable, and affordable public transportation	Lack of funding to afford transportation
	Perceptions and knowledge about disability	Attend talks, seminars as a tool to learn more about ones condition	Need more training on how to care for PWD and themselves	Many providers lack appropriate training in disability	People that design the city need to be aware of universal design
		Some professionals, including health and rehabilitation, lack of appropriate knowledge on disability and accessibility	Some rehabilitation providers do not have the training to appropriately work with PWD	Rehabilitation is not seen as inclusive by policy and decision makers	PWD and their families lack interest in learning
		Mistrust in medical personnel	Mistrust in medical system	Many PWD and caregivers do not adhere to the programs because lack of interest	PWD need training in rights and self-advocacy
				PWD and their families only identify as right-holders and not duty-bearers	Awareness on appropriate assistive technology is needed
	Navigating the system	Services constantly denied requiring legal appeal	Legal appeal required in many instances to access rehabilitation services	Disability is not a priority for policy makers	Legal appeal required to access services
		Pathways to access services are not clear	Services are insufficient	Lack of continuity in public programs and strategies	Lack of coordination between programs
			Pathways to access services are not clear	Lack of public funding for sport, art, and recreation	Non-existent care pathway
Participation in the community		Leisure and recreation participation most mentioned	Leisure and recreation participation most mentioned	Lack of interest and commitment from PWD and their families limit their community participation	Employment necessary to improve the quality of life of PWD and their families
		Education and employment important to social participation and to raise awareness on disability			Education is key to improve participation
					Sports as tools that teach independence, responsibility, and commitment
					Need for PWDs to take leadership roles

### Meaning of rehabilitation

Rehabilitation was conceptualized differently across participant groups. For PWDs, rehabilitation is focused on restoring function and includes physical therapy, mental health, practicing a sport, and accessing education. Rehabilitation was also viewed as necessary to overcome fear of leaving their house and needed to improve their lives. The value of rehabilitation is demonstrated through interaction with peers that have been successful with physical rehabilitation (they can do better). Similarly, for caregivers, rehabilitation related to regaining function and revolved around physical and psychological therapy, as well as the use of assistive technologies that improve the physical abilities of a PWD. Through rehabilitation comes acceptance of the disability. Most importantly, rehabilitation also meant less work for caregivers, as the PWD would be able to perform tasks without their help. Some caregivers also recognized the detrimental effect of over doing things for the PWD, which interfered in the rehabilitation process.

For professionals, rehabilitation was a strategy to promote independence for both the PWD and their caregiver, which should be tailored to individual needs. Professionals viewed independence not only as the ability to perform activities without help, but also the capability of a PWD to make decisions about their own life. Moreover, rehabilitation is viewed as a multilevel and multi sectorial process where professionals, families, and communities work alongside to achieve progress. From their perspective rehabilitation should include not only physical therapy, but assistive technology, education, employment training, sports, arts and recreation, where caregivers and families are also engaged. Other stakeholders had a similar perspective as rehabilitation professionals, seeing rehabilitation in most cases as a multilevel / multi sectorial process that “frees” the person with disability and their family. However, some professionals and other stakeholders still perceive rehabilitation as a strategy to go back to where the person was before an injury. See Additional file 2 for testimonies that illustrate this theme.

### Challenges to access comprehensive rehabilitation services

Three sub-themes emerged in the challenges to access services: barriers to personal mobility, perceptions and knowledge on disability, and navigating the system. See Additional file 3 for examples of testimonies illustrating each of the sub-themes.

### Barriers to personal mobility

All participants agreed on the challenges related to personal mobility. First, PWDs homes are inaccessible: they live in high floors without elevators, have small bathrooms or need to climb stairs to enter the home. All of these barriers significantly limit independence at home and the ability to leave the home. Second, lack of accessible and affordable public transportation posed a constant challenge to access available rehabilitation services and participate in the community. PWDs, rehabilitation professionals, and other stakeholders recognized that improvements have been made in the public transportation system: the Metro has stair platforms and there are buses with platforms for people with mobility impairments. However, they also acknowledged that the stair platforms are not always in working condition, not all buses have platforms, and the frequency in which buses with the platform circulate is unknown. In consequence, people often need to use taxis for transportation which is significantly more costly, and taxi drivers are not always willing to take a wheelchair in their car. Not having means to afford transportation was reported as one of the main causes for not accessing rehabilitation services and limiting participation in the community.

Moreover, all participants reported that public places (schools, universities, restaurants, movie theaters, sports facilities, etc.) were not accessible as many had stairs without ramps or alternative elevators, ramps were too steep, or bathrooms were too small. Several participants, across all groups, mentioned that sidewalks are inaccessible, forcing people to travel on the street increasing the risk of road accidents, plus the hilly condition of the city. The combination of the aforementioned challenges resulted in the PWDs needing an aid to be able to go outside of their home, increasing the cost of participation to account for the aids time and transportation.

Some PWDs, rehabilitation professionals, and other stakeholders discussed the negative impact of not having access to an appropriate wheelchair or prosthetic device that meets user’s needs, or the training on how to appropriately use them.

### Perceptions and knowledge about disability

Perceptions and knowledge about disability emerged as a challenge to access resources by all the participants. PWDs claimed the need to be acknowledged as individuals, without labels or classifications derived from their health condition. According to PWDs and caregivers, some rehabilitation professionals assume that people with the same health condition have the same functioning, ignoring what the PWD has to say about their needs and what they are able to do. Many caregivers referred that when their family member with a disability was discharged from hospitalization (after acquiring a disability) they did not receive enough training on how to face this new life. There is general mistrust in medical doctors, as initial diagnoses were given in a negative way and their outcomes in life have been more than what was initially predicted by doctors. Rehabilitation professionals, perceived similar experiences and described how the lack of training to work with PWDs results in poor communication, limiting the information professionals can share with PWDs and their families. From their perspective, it is common that PWDs do not receive information on what are the possibilities beyond the diagnosis. In addition, professionals perceived that current rehabilitation programs do not pay enough attention to sexuality and other ludic activities.

Some participants perceived that other professionals beyond the health care sector, such as architects and engineers, lack knowledge on disability and accessibility, which limits the universal access design of roads, buildings, houses and other physical spaces. Moreover, rehabilitation professionals and other stakeholders stated that publicly sponsored programs such as education, sports, and assistive technology are managed by people without experience working with PWDs. Appropriate training on disability and accessibility was identified as a general need. Both rehabilitation professionals and other stakeholders recognized that professionals are not the only ones responsible for promoting participation as they claimed that many PWDs and their caregivers are not interested in learning, do not to put enough effort, and expect the government and society to provide for them. In their perspective this leads to limitations for participation and disconnect between expectations and what the programs can offer. For example: many participants with disabilities were waiting for personnel in an employment center (e.g. public program) to find them a job and were not actively looking for one. Other stakeholders recognized that PWDs need better training about their rights and how to advocate for them, as well as improved self-awareness to envision their possibilities.

### Navigating the system

All acknowledged that the care pathway for PWDs is unclear for them and their families. Prescribed rehabilitation services may take several months to be approved by the health insurance and the number of office visits that are approved are often perceived as not enough, impacting the continuity of services. However, a contradicting view is explained by one PWD who criticized the lack of clear objectives in a rehabilitation process, suggesting users stay for years in the same rehabilitation program without a clear goal to strive for. Most PWDs and caregivers mentioned having to use legal resources because they were denied health and rehabilitation services, without receiving an explanation for the denial. Other stakeholders agreed that it is common for PWDs to legally appeal for services that are denied. In addition, caregivers shared that alternative services, such as equine therapy, are not covered by the health insurance and need to be paid out-of-pocket. One caregiver shared their concern with a bill that was being discussed at the moment of the interview that intended to cut out support for assistive technology by the healthcare system.

Last, rehabilitation professionals manifested that disability is not a priority in the public policy agenda, which affects the continuity of the public programs that are in place, making them dependent on the governor in office. Along the same lines, there is lack of governmental funding for sports (including adaptive equipment), artistic, and recreational programs. Other stakeholders indicated that there is a lack of articulation between programs and resources.

### Community participation

PWDs view participation as fundamental throughout the rehabilitation process. According to their experiences, education and employment are important because it engages them with social activities. Other stakeholders talked about the importance of employment to improve the quality of life of PWDs and their family environment, and the key role of education in improving participation. Nonetheless, some PWDs and other stakeholders mentioned that companies prefer not to hire people with disabilities due the legislation that protects PWDs from being fired due to their disability. See Additional file 4 for examples of testimonies that explore community participation.

Several PWDs mentioned activities they enjoy in their communities, mostly related to leisure and recreational activities (sports, arts, socializing with friends and family). Caregivers are supportive of these types of activities. PWDs, rehabilitation professionals, and other stakeholders also recognized the importance of sports to improve independence and create a sense of responsibility and commitment. Rehabilitation professionals and other stakeholders also stated that lack of interest and commitment from PWDs and their families limit their own community participation.

PWDs believe they can be influential by raising awareness on disability and advocating to make their communities more accessible. Another important topic raised discussed by PWDs, professionals, and other stakeholders, is the need for PWDs to lead initiatives related to disability. One PWD was very critical in stating that most disability-related programs do not hire PWDs.

## Discussion

Our findings provide evidence of the challenges that perpetuate the inequalities in access to rehabilitation services in a community of PWDs in Colombia. Despite de recent changes in the country’s legal framework, there is still significant disconnect between the needs of PWDs and the perspectives of other stakeholders. For instance, a national decree enacted in 2003, mandated accessibility for all new public buses starting in 2005 [31]. Our results indicate that although some improvements have been made, there is lack of consistency in the provision of accessible transportation. Public understanding and awareness of the needs of PWDs, lack of political will at the local level, as well as limited resources could be factors that contribute to the slow implementation of these policies especially outside of the capital cities.

Lack of consensus on the meaning of rehabilitation across PWDs and stakeholders, limits the efficacy of advocacy efforts to achieve equitable access to these services. It is also an indication that many still understand disability from the medical model: the individual with a disability has something wrong that needs to be fixed [32]. Previous work conducted in Bogota (before the National Disability Law) also reported lack of consensus between leaders with disability, policy makers, caregivers, inclusive education professionals, academics, and other professionals on their concept of the meaning of rehabilitation and the general belief that it relies solely on the health sector [25]. The outdated perceptions and knowledge about disability observed in our participants indicate that the mainstream culture continues to be in the medical model of disability [32], contributing to inequity in access to rehabilitation services that results in participation restrictions. A good example to provide some insight is the process to officially certify disability in Colombia. Even though the ICF framework is almost 20 years old [11], the first regulation in Colombia to certify disability based on the ICF was recently enacted in 2018 [33]. There is evidence to support that professional who are unaware of the capabilities and rights of PWDs become a hurdle [20] as they fail to refer people to the services that they need [34]. Attitudinal barriers of providers have also been reported in rural South Africa, Uganda, and in Colombia [35–37].

One potential strategy to foster a paradigm shift is through the explicit incorporation of disability in the curriculum of all levels of education, interventions can be developed to train professionals to work with PWDs, becoming facilitators instead of obstacles to participation [38, 39]. Higher education institutions have the responsibility to ensure that professionals joining the workforce, not only those in the health professions, are aware of the local and global disability policies incorporating a human rights-based approach [19, 25, 40]. Larger strives are needed to raise awareness on disability as part of human diversity and a human rights issue as mandated in article 4 of the CRPD [4]. A call for action is needed to develop stronger public-private collaborations that promote awareness on disability, with a rights-based approach, and comprehensive rehabilitation [8]. In synthesis, persistent understanding of disability from the medical model poses a risk to the acceptability factor of equitable access to rehabilitation [6].

Even though many participants defined rehabilitation from the perspective of the medical model of disability, when referring to the challenges to access services, almost all were placed outside of the individual (i.e. social model of disability) [41]. Specifically, critical contextual factors included inaccessibility at home an in physical spaces (including transportation), lack of appropriate assistive technology, cultural beliefs and public’s knowledge about disability, and non-existent care path [11]. These challenges posed by contextual factors to achieve health for all are recognized by the United Nations [8]. For example in Africa lack of transportation, availability of services, inadequate equipment or drugs, and costs as the main barriers for access to health care among PWDs [42–44]. In Brazil, lack of physical accessibility of hospitals for PWD has been reported as an important barrier to be addressed [45]. In Tunja, Colombia, use of taxi cabs by PWDs was reported as being 2.6 times higher than others without disability due to the lack of accessible buses [46]. A study in Chile, recently called for improvement in public transport and metro system accessibility for PWDs [47]. Improving physical accessibility is not only a requirement to improve equity in access to care, but also a pre-requisite for social participation as PWDs in living in challenging contexts require extensive planning to go somewhere because of the barriers in the built-environment [48]. Contextual factors increase the cost of participation and healthcare due to the need for personal assistance, assistive technology, and private transportation to go to a healthcare facility [4, 35, 49–51]. Ensuring personal mobility with independence for PWDs is a human right in the CRPD (article 20) [4] and is prerequisite to achieve the Sustainable Development Goals [8, 52, 53].

The lack of a formal care pathway for PWDs and their families negatively contributes to their ability to timely access comprehensive rehabilitation services. The Colombian Disability Law, and later the National Disability Public Policy in 2013, mandated the Ministry of Health to provide a care pathway model for comprehensive rehabilitation, with clear directions on how different sectors should articulate and collaborate [17, 18]. This model is not published as of yet. Lack of coordination in the provision of care affects availability of adequate resources necessary to have appropriate access to comprehensive rehabilitation [6, 8]. Engagement of PWDs, communities and stakeholders is needed for the development and implementation of a model that addresses the needs of those with disability. The National Disability System should prioritize the development of this model [54], including a clear monitoring and evaluation strategy.

Participation in activities that one enjoys, has a positive impact on the quality of life [48]. There is a disagreement between the perspective of professionals and other stakeholders on the level of participation, leadership and self-advocacy ability of PWDs and their families and that of PWDs. Those on the provider side claim that PWD still need more commitment and engagement with participation. PWDs state the existence of barriers to reach and participate in leadership positions, even for disability-related programs which according to the disability-rights movement should be led by PWDs “nothing about us, without us” [55]. The high unemployment rates of PWD in Colombia support this claim [24]. Deliberated efforts to promote qualified individuals with disabilities to leadership positions must be taken. The recently enacted public employment regulation quotas for employees with disabilities may have a positive impact in this aspect [56].

By engaging stakeholders representing different sectors of the community, we were able to characterize the diversity in their perspectives on comprehensive rehabilitation, and identify actionable gaps, as our sample size allowed us to reach saturation among the main themes of interest. We acknowledge that our purposive sample may select PWDs, caregivers, rehabilitation professionals, and other stakeholders who are more engaged with services and the community. However, through our sampling strategy we ensured that our sample would represent diverse experiences by identifying participants who where heterogeneous in their physical disability, gender, socioeconomic status, education level, and role. Although the majority of our informants were women, this is consistent with gender differences in health and social services professions [57, 58] and caregiving roles [59–61]. It is important to note that at the population level there are more men with physical disabilities within the age range of our study [24]. Other studies have reported that women participate more in research than men [62, 63], potentially indicating that better rapport was built with women. The income distribution in our sample is similar to that reported for the Colombian population with disability, with 80% living in low-income households [24].

Even though this study depicts the experience of the city of Envigado, the challenges could be transferable to other contexts with an evolving legal disability framework. One of our limitations is that the perspectives presented in this work only those of people with physical disabilities. To better inform inclusive strategies aimed at reducing inequalities in rehabilitation and health for all PWDs, the perspectives of people with other types of impairments must be taken into consideration. For instance, for people who are deaf communication may be one of the most significant barriers to accessing health services with equity [64], while for people with visual impairment and intellectual disabilities, written information (prescriptions, home plans, referrals, etc.) may become important barriers [8, 65]. Last, experiences of the military forces –as there is a significant number of members with disabilities due to the conflict– could also be explored since the health care regulations that cover them are different than for the civilian population target in this study [66]. In addition, there were themes for which we did not find saturation but may be relevant to equity in health such as sexuality of PWDs. See Additional file 5 to access the manuscript in Spanish.

## Conclusions

Participation in the community is the ultimate aim of comprehensive rehabilitation. Significant gaps in the translation of the national disability framework such as outdated disability constructs, ongoing architectural and mobility challenges, lack of trained personnel, and difficulty to understand and navigate the system, limit access to comprehensive rehabilitation programs. By identifying actionable gaps, communities can become empowered and lead advocacy efforts to achieve equitable access to comprehensive rehabilitation for PWDs and their families.

## Additional files

Additional file 1: Semi-structured interview guides in Spanish per type of participant. (DOCX 142 kb)

Additional file 2: Testimonies depicting the meaning of rehabilitation by type of participant. This additional file includes a table with the testimonies by type of participant for their meaning of rehabilitation. (DOCX 124 kb)

Additional file 3: Testimonies illustrating the challenges to access comprehensive rehabilitation services. This additional file includes a table with the testimonies by type of participant per sub-theme: barriers to personal mobility, perceptions and knowledge on disability, and navigating the system. (DOCX 124 kb)

Additional file 4: Testimonies illustrating the community participation theme that emerged from participants experiences. This additional file includes a table with the testimonies by type of participant. (DOCX 122 kb)

Additional file 5: manuscript in Spanish. (DOCX 110 kb)

## Abbreviations

CRPD: Convention on the Rights of Persons with Disabilities
ICF: International Classification of Functioning, Disability, and Health
PWD: People with Disabilities
WHO: World Health Organization
