# Supplementary material for: Experiences with rehabilitation and impact on community participation among adults with physical disability in Colombia: perspectives from stakeholders using a community based research approach
Source: Int J Equity Health. 2019 Jun 3;18:18. doi: 10.1186/s12939-019-0923-4 (PMC6545726; doi:10.1186/s12939-019-0923-4)
Supplement: Supplementary file 1 — Semi-structured interview guides in Spanish per type of participant. (DOCX 142 kb) [file 12939_2019_923_MOESM1_ESM.docx]

Additional file 1

Semi-structured interview guides in Spanish per type of participant.

# Guía para entrevistas usuarios de ALFIME

Código participante______________________________

*Esta descripción se hará después de que el entrevistador se presente, se haga el proceso del consentimiento informado y el participante acepte continuar con la entrevista.*

Gracias por aceptar participar en esta entrevista que durará alrededor de una hora. Lo que queremos lograr con esta entrevista es conocer sus experiencias con la rehabilitación y lo que percibe sobre las necesidades en este sentido para usted, su familia y las personas que comparten con usted historias similares en términos de la discapacidad. Es muy importante su punto de vista y sus experiencias con el fin de fortalecer a ALFIME tanto internamente como en la relación con otras instituciones externas. Una entrevista es una conversación, siéntase libre de usar cualquier expresión y de contarme lo que quiera. Recuerde que esta conversación es confidencial, es decir, nadie sabrá que usted fue la persona que lo contó ¿Tiene alguna pregunta antes de empezar?

Independencia y autonomía

¿Me puede contar cómo es un día normal para usted desde que se levanta hasta que se acuesta?

¿Para qué actividades requiere usted ayuda de otra persona?

- Sondeo: ¿Cómo se siente de necesitar ayuda de otra persona para realizar sus actividades cotidianas?

¿En qué aspectos de su vida puede realizar actividades sin ayuda de otra persona?

- Sondeo: ¿Cómo se siente de poder realizar actividades sin ayuda de otra persona?

Percepción de discapacidad

Usted vive con XXX condición, ¿cómo se siente de vivir con esta condición?

¿qué tanto considera que esta condición es una discapacidad?

¿qué piensa acerca de que otras personas consideren su condición XXX como una discapacidad?

Sondeo: ¿usted nació con esta condición o la adquirió en algún punto en su vida?

Sondeo, si nació con la condición:

¿Cómo ha sido para usted vivir con esta condición toda su vida?

Sondeo, si la adquirió en algún momento de su vida:

¿cómo incorporó su condición XXX en su vida?

¿qué cosas cambiaron?

- ¿qué cosas siguieron igual?

Recursos de rehabilitación: disponibles, necesidades insatisfechas y prioridades

¿Me puede contar qué servicios y/o programas de rehabilitación conoce que existen?

¿Me puede contar qué se debe hacer para asistir a un servicio o programa de rehabilitación?

- Sondeo: ¿Me puede contar a qué servicio y/o programa de rehabilitación asiste?
- Sondeo: ¿Quién paga por estos servicios?

¿Me puede dar un ejemplo de alguna vez en la que necesitaba ir a un servicio y no lo pudo hacer?

Percepción de rehabilitación

¿Me puede contar cómo fue su experiencia la última vez que visitó un servicio de rehabilitación?

¿Me puede contar cómo le parece que la rehabilitación afecta su vida?

Procesos ciudadanos

¿Me puede contar qué piensa sobre poderle ayudar a la comunidad?

¿En qué actividades o iniciativas está involucrado que impacte la comunidad?

¿Me podría contar qué mecanismos conoce que existan para hacer cambios en la comunidad?

¿Qué cambio considera que deben suceder en la comunidad?

- Sondeo: ¿Considera que está en la capacidad de contribuir a que sucedan estos cambios?

Ha sido muy interesante escuchar sus experiencias. Para terminar, ¿quisiera comentar algo adicional sobre algún tema que tratamos hoy o sobre esta entrevista? Agradezco mucho su tiempo. Le reitero que todo lo que conversamos hoy es confidencial, no se sabrá que fue usted quien lo dijo.

# Guía para entrevista con cuidadores de usuarios de ALFIME

Código participante______________________________

*Esta descripción se hará después de que el entrevistador se presente, se haga el proceso del consentimiento informado y el participante acepte continuar con la entrevista.*

Gracias por aceptar participar en esta entrevista que durará alrededor de una hora. Lo que queremos lograr con esta entrevista es conocer sus experiencias con la rehabilitación y lo que percibe sobre las necesidades en este sentido para usted, su familia y las personas que comparten con usted historias similares en términos cuidador o tutor de una persona discapacidad. Es muy importante su punto de vista y sus experiencias con el fin de fortalecer a ALFIME tanto internamente como en la relación con otras instituciones externas. Una entrevista es una conversación, siéntase libre de usar cualquier expresión y de contarme lo que quiera. Recuerde que esta conversación es confidencial, es decir, nadie sabrá que usted fue la persona que lo contó ¿Tiene alguna pregunta antes de empezar?

Independencia y autonomía

¿Me puede contar cómo es un día normal para usted desde que se levanta hasta que se acuesta?

¿Para qué actividades requiere la persona que usted cuida que le ayude?

- Sondeo: ¿Cómo se siente tenerle que ayudar en las actividades cotidianas a la persona que cuida?
- Sondeo: ¿Me puede contar sobre alguna ocasión en que ha sido difícil ayudarle a la persona que cuida?
- Sondeo: ¿Me puede contar sobre alguna ocasión en la que ha tenido que pedirle ayuda a otra persona para que le ayude a cuidar a la persona que cuida?
  - ¿Cómo se siente cuando le tiene que pedir ayuda a otra persona para que le ayude a cuidar a la persona que cuida?

¿En qué aspectos de la vida la persona que cuida puede realizar actividades sin ayuda suya o de otra persona?

- Sondeo: ¿Cómo se siente que la persona que cuida pueda realizar actividades sin su ayuda o de otra persona?

Percepción de discapacidad

La persona que usted cuida vive con XX condición, ¿cómo se siente que ella/él viva con esta condición?

¿Qué tanto considera que esta condición es una discapacidad?

¿Qué piensa acerca de que otras personas consideren la condición XXX de la persona que cuida como una discapacidad?

Sondeo: ¿la persona que cuida nació con esta condición o la adquirió en algún punto en su vida?

Sondeo, si nació con la condición:

¿Cómo ha sido para usted que la persona que cuida viva con esta condición toda su vida?

Sondeo, si la adquirió en algún momento de su vida:

¿Cómo incorporó la condición XXX de la persona que cuida en su vida?

¿Qué cosas cambiaron para la persona que cuida?

¿Qué cosas cambiaron para usted?

- ¿Qué cosas siguieron igual para la personas que cuida?
- ¿Qué cosas siguieron igual para usted para usted?

Recursos de rehabilitación: disponibles, necesidades insatisfechas y prioridades

¿Me puede contar qué servicios y/o programas de rehabilitación conoce que existen?

¿Me puede contar qué se debe hacer para asistir a un servicio o programa de rehabilitación?

- Sondeo: ¿Me puede contar a qué servicio y/o programa de rehabilitación asiste la persona que cuida?
- Sondeo: ¿Quién paga por estos servicios?

¿Me puede contar qué piensa acerca del papel/rol que cumple usted en el uso de los servicios de rehabilitación de la persona que cuida?

¿Me puede dar un ejemplo de alguna vez en que la persona que cuida necesitaba ir a un servicio y no lo pudo hacer?

¿Me puede contar qué servicios o programas conoce que existen para cuidadores como usted?

- Sondeo: ¿Quién paga por estos servicios?

¿Qué cosas le gustaría hacer que no puede hacer porque tiene que cuidar a su familiar?

- Sondeo: ¿En qué usaría usted su tiempo si pudiera contar con ayuda para el cuidado de la persona a quien usted cuida?

Percepción de rehabilitación

¿Me puede contar cómo fue su experiencia la última vez que visitó un servicio de rehabilitación junto con la persona que cuida?

¿Me puede contar cómo le parece que la rehabilitación afecta la vida de la persona que cuida?

- Sondeo: Ahora, ¿me puede contar cómo le parece que la rehabilitación afecta su vida como cuidador?

Procesos ciudadanos

¿Me puede contar qué piensa sobre poderle ayudar a la comunidad?

¿En qué actividades o iniciativas está involucrado que impacte la comunidad?

¿Me podría contar qué mecanismos conoce que existan para hacer cambios en la comunidad?

¿Qué cambio considera que deben suceder en la comunidad?

- Sondeo: ¿Considera que está en la capacidad de contribuir a que sucedan estos cambios?

Ha sido muy interesante escuchar sus experiencias. Para terminar, quisiera comentar algo adicional sobre algún tema que tratamos hoy o sobre esta entrevista. Agradezco mucho su tiempo.

# Guía para entrevistas profesionales de ALFIME

Código participante______________________________

*Esta descripción se hará después de que el entrevistador se presente, se haga el proceso del consentimiento informado y el participante acepte continuar con la entrevista.*

Gracias por aceptar participar en esta entrevista que durará alrededor de una hora. Lo que queremos lograr con esta entrevista es conocer sus experiencias con la rehabilitación y lo que percibe sobre las necesidades en este sentido para las personas con discapacidad. Es muy importante su punto de vista y sus experiencias con el fin de fortalecer a ALFIME tanto internamente como en la relación con otras instituciones externas. Una entrevista es una conversación, siéntase libre de usar cualquier expresión y de contarme lo que quiera. Recuerde que esta conversación es confidencial, es decir, nadie sabrá que usted fue la persona que lo contó ¿Tiene alguna pregunta antes de empezar?

Independencia y autonomía

¿Me podría contar qué es para usted la independencia?

¿Me podría contar qué es para usted la autonomía?

Percepción de discapacidad

¿Me podría describir qué es para usted una persona con discapacidad?

¿Cómo se puede promover la independencia entre las personas con discapacidad?

¿Cómo se puede promover la autonomía entre las personas con discapacidad?

Recursos de rehabilitación: disponibles, necesidades insatisfechas y prioridades

¿Me puede contar cómo se ha capacitado para brindar servicios a las personas con discapacidad?

¿Me puede contar con qué recursos cuenta para brindar los servicios con los que trabaja?

¿Me podría contar qué considera usted que necesita para mejorar el servicio que provee?

¿Me puede contar qué servicios y/o programas de rehabilitación conoce que existen?

¿Me puede contar qué se debe hacer para asistir a un servicio o programa de rehabilitación?

- Sondeo: ¿Quién paga por estos servicios?

¿Me puede contar con qué dificultades conoce que se encuentran las personas con discapacidad para acceder a servicios o programas de rehabilitación?

- Sondeo: ¿Me puede dar un ejemplo de alguna vez en la que un usuario de Alfime necesitaba ir a un servicio y no lo pudo hacer?

¿Me puede contar cuál piensa que es el papel que cumple el cuidador en el uso de servicios de rehabilitación de una persona con discapacidad?

¿Me puede contar qué servicios conoce que existen para los cuidadores de personas con discapacidad?

¿Me podría contar cuáles son las necesidades de la comunidad de las personas con discapacidad?

Percepción de rehabilitación

¿Me puede contar cómo le parece que la rehabilitación afecta la vida de las personas con discapacidad?

¿Me puede contar cómo le parece que la rehabilitación afecta la vida de las personas cuidadoras de personas con discapacidad?

Procesos ciudadanos

¿Me puede contar qué piensa sobre poderle ayudar a la comunidad?

¿En qué actividades o iniciativas está involucrado que impacte la comunidad?

¿Me podría contar qué mecanismos conoce que existan para hacer cambios en la comunidad?

¿Qué cambio considera que deben suceder en la comunidad?

- Sondeo: ¿Considera que está en la capacidad de contribuir a que sucedan estos cambios?

Ha sido muy interesante escuchar sus experiencias. Para terminar, quisiera comentar algo adicional sobre algún tema que tratamos hoy o sobre esta entrevista. Agradezco mucho su tiempo.

# Guía para entrevistas: Otros actores

Código participante______________________________

*Esta descripción se hará después de que el entrevistador se presente, se haga el proceso del consentimiento informado y el participante acepte continuar con la entrevista.*

Gracias por aceptar participar en esta entrevista que durará alrededor de una hora. Lo que queremos lograr con esta entrevista es conocer sus experiencias con la rehabilitación y lo que percibe sobre las necesidades en este sentido para las personas con discapacidad. Es muy importante su punto de vista y sus experiencias con el fin de fortalecer a ALFIME tanto internamente como en la relación con otras instituciones externas. Una entrevista es una conversación, siéntase libre de usar cualquier expresión y de contarme lo que quiera. Recuerde que esta conversación es confidencial, es decir, nadie sabrá que usted fue la persona que lo contó ¿Tiene alguna pregunta antes de empezar?

Independencia y autonomía

¿Me podría contar qué es para usted la independencia?

¿Me podría contar qué es para usted la autonomía?

Percepción de discapacidad

¿Me podría describir qué es para usted una persona con discapacidad?

¿Cómo se puede promover la independencia entre las personas con discapacidad?

¿Cómo se puede promover la autonomía entre las personas con discapacidad?

Recursos de rehabilitación: disponibles, necesidades insatisfechas y prioridades

¿Me puede contar qué servicios y/o programas de rehabilitación conoce que existen?

¿Qué apoyo o programas de rehabilitación ofrece la organización para la que trabaja?

¿Me puede contar qué se debe hacer para asistir a un servicio o programa de rehabilitación?

- Sondeo: ¿Quién paga por estos servicios?

¿Me podría contar con qué dificultades conoce que se encuentran las personas con discapacidad para acceder a servicios o programas de rehabilitación?

- Sondeo: ¿Me puede dar un ejemplo de alguna vez que una persona con discapacidad necesitaba ir a un servicio y no lo pudo hacer?

¿Me puede contar cuál piensa que es el papel que cumple el cuidador en el uso de servicios de rehabilitación de una persona con discapacidad?

¿Me puede contar qué servicios conoce que existen para los cuidadores de personas con discapacidad?

¿Me podría contar cuáles son las necesidades de la comunidad de las personas con discapacidad?

Percepción de rehabilitación

¿Me puede contar cómo le parece que la rehabilitación afecta la vida de las personas con discapacidad?

¿Me puede contar cómo le parece que la rehabilitación afecta la vida de las personas cuidadoras de personas con discapacidad?

Procesos ciudadanos

¿Me puede contar qué piensa sobre poderle ayudar a la comunidad?

¿En qué actividades o iniciativas está involucrado que impacte la comunidad?

¿Me podría contar qué mecanismos conoce que existan para hacer cambios en la comunidad?

¿Qué cambio considera que deben suceder en la comunidad?

- Sondeo: ¿Considera que está en la capacidad de contribuir a que sucedan estos cambios?

¿Me podría contar cómo su visión de las personas con discapacidad influye en las decisiones que toma su organización?

Ha sido muy interesante escuchar sus experiencias. Para terminar, quisiera comentar algo adicional sobre algún tema que tratamos hoy o sobre esta entrevista. Agradezco mucho tu tiempo.
