# Supplementary material for: Experiences with rehabilitation and impact on community participation among adults with physical disability in Colombia: perspectives from stakeholders using a community based research approach
Source: Int J Equity Health. 2019 Jun 3;18:18. doi: 10.1186/s12939-019-0923-4 (PMC6545726; doi:10.1186/s12939-019-0923-4)
Supplement: Supplementary file 2 — Testimonies depicting the meaning of rehabilitation by type of participant. This additional file includes a table with the testimonies by type of participant for their meaning of rehabilitation. (DOCX 124 kb) [file 12939_2019_923_MOESM2_ESM.docx]

Additional file 2

Testimonies depicting the meaning of rehabilitation by type of participant.

| **People with disabilities** | **Caregivers** | **Rehabilitation Professionals** | **Other stakeholders** |
| --- | --- | --- | --- |
| “...They have helped me a lot and I have learned a lot. Many things about being independent. That I can’t get sad for the things I can’t do, that I am in fact able to do them, that I am going to shower by myself, that I can go to a park by myself. I have learned that I need to stop being afraid about getting on the ramps” | “rehabilitation is a very good thing to move on and helps a lot, because you don’t know, when you don’t have experience and you get it [disability], then you don’t know what to do. It is very good because it opens your eyes and you learn by during in the way” | “We all have physical rehabilitation in our heads, and what happens with the other rehabilitation? Independent living process do talk about it all…I think that rehabilitation based in autonomy and independence is the only one that will allow you to live as natural as possible” | “when we speak of rehabilitation and habilitation, we do it from an integral concept beyond the clinical aspect….we work in all the human dimensions: family, work, education, health” |
| “With rehab and therapy life is easier, without therapy one cannot improve, then therapy is really good.” | “…We hope that this rehabilitation that is been done on him [brother with physical disability] works and later he goes back…we are aware that he won’t recover 100%, but at least 80% would be great. The problem is that his hand does not want to work…he says if my hand would help me, everything would be different…” | “…from rehabilitation, I think it is to assist, to support….but I believe that there are more efficacious ways like art, sports, I think people will rehabilitate better. With the work rehabilitation, there is no joy…but, it seems that for people it is ok, that it is the way to go, that it is very important” | “Rehabilitation is when you had something and then lost it due to an event and then I acquire it back. Habilitation is when you are born with something, you can do normal things that are done with development.” |
