# Supplementary material for: Experiences with rehabilitation and impact on community participation among adults with physical disability in Colombia: perspectives from stakeholders using a community based research approach
Source: Int J Equity Health. 2019 Jun 3;18:18. doi: 10.1186/s12939-019-0923-4 (PMC6545726; doi:10.1186/s12939-019-0923-4)
Supplement: Supplementary file 3 — Testimonies illustrating the challenges to access comprehensive rehabilitation services. This additional file includes a table with the testimonies by type of participant per sub-theme: barriers to personal mobility, perceptions and knowledge on disability, and navigating the system. (DOCX 124 kb) [file 12939_2019_923_MOESM3_ESM.docx]

Additional file 3

Examples of testimonies depicting the challenges to access comprehensive rehabilitation

|  | **People with disabilities** | **Caregivers** | **Rehabilitation Professionals** | **Other stakeholders** |
| --- | --- | --- | --- | --- |
| **Barriers to personal mobility** | “...I have found jobs...far, for which I need to take the Metro...but you know, you have to be responsible, I can’t call and say...hey, I can’t go today because the Metro stair platform is not working...I could take a taxi with platform, but they are expensive, so it is not worth it either...so basically, I don’t work because of the transportation” | “…sometimes I have not been able to take him [brother with disability] to therapy because we don’t find who drives the car …so my son asks us when do we have therapy so he takes time from work to transport us…in taxi is difficult…he gets rigid when you try to move him…” | “If the person doesn’t have the wheelchair they need and he/she doesn’t know how to use it, for example pop the casters to go over a curb without a curb-cut, they won’t be able to go out independently” | “..a fundamental field it the physical urban environment, in a way that it allows people to go out with freedom, autonomy, safety y allows them to use it with their own characteristics…how through accessible spaces we can achieve the inclusion” |
|  | “..I can’t walk right now. I live in a high raise apartment without elevator. I have to ask two people to help me go out and come back in, people are not available all the time…I go out once a week…” | “…we can’t take his power wheelchair [son with disability] in a car, it is like a truck, very heavy...you can’t transport it…he had to go out and come back on that [driving the power wheelchair]…” | “Many universities are not adapted [accessible] and if I have class in a third floor, I talk to the director, he could say that it is my problem that the class has to be there, then you don’t go back” | “…there [in Spain] access to wheelchairs is protected by the health care system…here you have to wait three years, even to wait to die, so you can get one…” |
| **Perceptions and knowledge on disability** | “...I had a bad experience [with physiotherapy]...I am not a person that says NO at first...so if you tell me do to something, I try. One physiotherapist told me to transfer to my wheelchair, I tried and I couldn’t...then she said: ‘look at this other person, she has the same condition as you and can do it’...that is why I don’t go to therapy anymore, you can’t tell people to do what they know they can’t do...” [PWD]. | “'…we have to be aware of the meetings they hold [Alfime], they teach us how to handle them…sometimes wanting to do good, we harm…you feel pity and end up doing many things for them…but no, we have to let them do it.” | “..for people that acquire a disability or that are born with a disability, the health care sector is the first to see them...and they say, go home, but I believe they should be the first ones to open possibilities and not closing the door...people go home thinking the only resource is the doctor. We continue to have a medical concept of disability....” | “…there is a law that gives tax brakes to companies that hire PWD…we have to work not only with the companies but with PWD. There are many [PWDs] that think that because they have a disability, they are an obligation to society [society must provide everything to them], they exclude themselves. |
|  | “they [my parents, uncle] don’t let me out [to run errands] by myself…because I’m the only one who has this condition, they all protect me to much…all are the girl, the girl….is like I didn’t become a grown up” | “...since she started high school I begged the school to build ramps, she had classes in the first floor and then had to go to the third...I begged for the ramp until she graduated but there was no God to make them [school leaders] do it...I proposed several alternatives: that I would build it, or pay a person to build it...it was very painful...they never [school leaders] understood the need and she was always carried [to the third floor] up the stairs...” | “'People think that the problem is that you don’t walk…but, the rest of the consequences? …imagine you are laying with your husband, you wake u and you peed, you have your period. Your husband may be lovely…but, how does this make you feel? If you are in a taxi and you pee or poop, taxi drivers may be nice and I can get home and get cleaned…but all of what this means mentally and emotionally, my tolerance to frustration for the next time out..” | “..now there are campaigns to make everything accessible, plus there is a law…but you see that they [engineers] build ramps…but try to go up the ramp. They do them just to meet the law, not with common sense…” |
|  | “…what happens is that the sector [disability] does not believe [in people with disabilities] and the efforts are only assistance. Disability equals impossibility, there is nothing to take” |  | “I think we should prepare them in the university [physical education students]…even if they are going to work in a conventional school, they will have students with disabilities…what they do now is ask the student [with physical impairment] to write about what is basket ball” | “…before I started working in the [disability] field, I don’t know if I felt fear, pity, or indifference, I was unable to look into the eys of a PWD, I didn’t even feel the confidence to offer help. After I started working in the field, now I see them normal…to me it doesn’t make a difference whether they have a disability or not…this is what society is lacking…realizing that they [PWD] are also part of society…” |
| **Navigating the system** | “When I got here…I told them [ALFIME] I want to study! I wanted from before but I couldn’t because I needed transportation everyday. Here they told me I could study Saturday and Sunday. Then I went to the Secretary of Education…get a number to get the spot…I went and that is when I got [4^th^ and 5^th^ grade] in…” | “…I tell people do this, this, and this, go to the entity that oversees health services [Super salud] that in 5 days they resolve your problems…I give people in line [waiting for approvals/medications]…I have investigated how you do everything by yourself…the health insurance does everything very limited” | “People think that because they shower and get into the car, they are rehabilitated…and it is not like that. The thing is that what does the healthcare system do when you have an accident, they send you to a physiatrist and physiotherapist, yes, they should, but what about the other side? Well if you can´t do it, find a psychologist. “I can’t” is a very deep topic and society and people themselves can’t dimension its complexity…” | “…when a person has an accident, …and then has to use a wheelchair…therapeutic support is fundamental…we are leaving people alone and the healthcare system is not doing what they should which is medical and therapeutic support….we need to know that….assimilating this process is very hard, and harder if you are alone” |
|  | “..I got my power wheelchair without legal appeal, thank God...it was a miracle, because I didn’t have to legally appeal..” | “…he [son with disability] has been scored 4 times [labor capacity] but they don’t explain to you anything…it is worse when you go to the [disability] pension center…they don’t know what you are talking about…they [office personnel] even contradict themselves…they request you to bring paper work and then they realize they are the ones that should have that documentation internally …we are screwed..” | “…I see that things are very difficult and sad, government programs are very limited, end in November and restart in March…people are left without support this period …public programs have closed because the healthcare system is supposed to provide those services…but, go and see them…disability is not a national priority…” | '…when I acquire a disability it is fundamental to know what is my support network…you don’t receive a manual, there is no support to guide you…there should be strengthening and coordination …an active network ready to provide the services needed…” |
