# Supplementary material for: Experiences with rehabilitation and impact on community participation among adults with physical disability in Colombia: perspectives from stakeholders using a community based research approach
Source: Int J Equity Health. 2019 Jun 3;18:18. doi: 10.1186/s12939-019-0923-4 (PMC6545726; doi:10.1186/s12939-019-0923-4)
Supplement: Supplementary file 4 — Testimonies illustrating the community participation theme that emerged from participants experiences. This additional file includes a table with the testimonies by type of participant. (DOCX 122 kb) [file 12939_2019_923_MOESM4_ESM.docx]

Additional file 4

Testimonies illustrating the community participation theme that emerged from participants’ experiences.

| **People with disabilities** | **Caregivers** | **Rehabilitation Professionals** | **Other stakeholders** |
| --- | --- | --- | --- |
| “There are organizations of people with disabilities but with low advocacy power… none of the organizations are big or with power or funds” | “…when he [son with disability] graduated from high school he applied to three universities, including Envigado because it is close to where we live and it would have been a good option for going there. He also applied to a private university and got accepted there with a scholarship...he is there now, strugling with transportation but well, he is very happy stuying” | “…when they come [PWDs] you give them a plan, and they are the reflection of their family…they do not believe…there is lack of commitment, passive attitudes…they come with a hopeless medical report…so the family doesn’t assume the situation…they [PWD] don’t think in saying I can, I will do it…it is difficult for them to engage” | “…as we were talking before, the city’s program [promotes employment for PWD] was born from the initiative of the population that was captured in the city’s disability policy…they considered that there is a lack of offer for income.” |
| “ I really like it [the job], I was very happy, but it was very hard. I worked from 10pm-6am…I studied from 4pm-9:30pm…I really liked it, but the company closed” | “…one day Alfime exaplained to her [daughter with disability] that Boccia was a paralympic sport..she started training, did well, and got in the regional team…she had to quit…but she has preferred more to study…but for others that don’t like to study, this [practicing a sport] is the only thing that ignites their passion…” | “…few people have access Antioquia’s University [public], they have not been admitted…well, I know that about 5 years ago a guy with low vision was denied his opportunity to study music there…” | “it is easy to access sports, the problem is for PWD to actually go and practice sports. It is difficult because of resources; sometimes there is not a proper coach available. But, even when there is a coach, what do you do if you do not have means to get there…getting the coach to a closer location where everyone can go could be a way around this” |
