# Supplementary material for: Experiences with rehabilitation and impact on community participation among adults with physical disability in Colombia: perspectives from stakeholders using a community based research approach
Source: Int J Equity Health. 2019 Jun 3;18:18. doi: 10.1186/s12939-019-0923-4 (PMC6545726; doi:10.1186/s12939-019-0923-4)
Supplement: Supplementary file 5 — manuscript in Spanish. (DOCX 110 kb) [file 12939_2019_923_MOESM5_ESM.docx]

**Título: Las experiencias** de adultos con discapacidad física con la rehabilitación y su impacto en la participación den comunidad en Colombia: perspectivas de actores desde un estudio con enfoque basado en participación comunitaria

**Resumen**

**Antecedentes:** A pesar de representar 70 millones de personas en Latinomérica, el acceso a la rehabilitación integral y la participación en comunidad sigue siendo un reto para las personas con discapacidad (PCD) de la región. Con la Ley Estatutaria de Discapacidad, Colombia a logrado avances en el reconocimiento y disminución de algunas de las barreras que enfrentan las PCD, incluyendo el derecho a la rehabilitación integral. Sin embargo, el acceso es limitado con una desconexión importante entre las perspectivas de los diferentes actores y las necesidades de la población. Examinamos las percepciones con respecto al acceso a los servicios rehabilitación integral y a la participación de las PCD. Además, exploramos la perspectiva sobre las experiencias de las PCD de los cuidadores de PCD, profesionales de la rehabilitación y otros actores con experiencia en discapacidad. Nuestro objetivo era identificar brechas en la implementación de programas de rehabilitación integral y las barreras para accede a dichos recursos/servicios que pudiesen impactar la participación de las PCD.

**Métodos:** Estudio cualitativo desarrollado en el 2017. Los datos fueron recolectados de una muestra intencional de adultos con discapacidad física entre 18 y 44 años que habían recibido servicios de rehabilitación en una organización local. Además, tenían diferentes antecedentes y experiencias con discapacidad. El muestreo intencional fue también llevado acabo con cuidadores, profesionales de rehabilitación y los otros actores. Información sociodemográfica fue recolectada y entrevistas semi-estructuradas fueron llevadas acabo por un miembro del equipo investigador, grabadas, transcritas y analizadas utilizando análisis temático para identificar los temas principales relacionados con nuestro objetivo. Este estudio contó con el aval del comité de ética de la Universidad CES.

**Resultados:** 32 participantes fuereon entrevistados: ocho hombres, 42.1 ± 11.1 años de edad, and 44% (n=14) eran PCD. Tres temas principales fueron identificados entre todos los participantes: el significado de la rehabilitación, retos para acceder a servicios y participación. Dentro de los retos para acceder a servicios se identificaron tres subtemas: barreras para la movilidad personal, percepciones y conocimiento sobre discapacidad y navegar el sistema.

**Conclusión**: La rehabilitación es percibida principalmente por los participantes como rehabilitación funcional. Si los profesionales de la salud son capacitados en discapacidad y las personas con discapacidad están involucrados activamente en esteos programas, el enfoque podría cambiar hacia una rehabilitación integral en igualdad de condiciones que promueva la participación plena de las PCD.

**Palabras claves**

Discapacidad física, rehabilitación integral, participación comunitaria.

**Introducción**

Setenta millones de personas en Latinoamérica viven con una discapacidad, se observa una alta prevalencia en los más pobres y vulnerables [1]. La Comisión para América Latina y el Caribe estima que 12.6% de la población de la region tiene una discapacidad y se espera que incremente debido factores como el envejecimiento, las enfermedades no transmisibles, la pobreza, los conflictos armados y la violencia urbana y de género [2, 3]. La mayoría de los países en la región han ratificado la Convención de las Naciones Unidas de los Derechos de las Personas con Discapacidad (Convención); sin embargo, el acceso a la habilitación, rehabilitación y servicios de salud por parte de las personas con discapacidad (PCD) continua siendo un reto, limitando las oportunidades de participación [4, 5]. El acceso a los servicios es un concepto complejo que involucra factores como la disponibilidad, la accesibilidad física, la oferta adecuada, la asequibilidad económica y la aceptabilidad de la población [6]. Por ende, el acceso equitativo a la habilitación, rehabilitación y los servicios de salud depende de una compleja interacción entre factores en los niveles personales, comunitarios, contextuales y sistémicos [7]. Debido a que el acceso a estos servicios es crítico para satisfacer las necesidades de las PCD y promover su participación [8], investigadores en el campo lo han propuesto como un indicador de equidad [9, 10].

La Clasificación Internacional del Funcionamiento, la Salud y la Discapacidad (CIF) define la discapacidad como un concepto que evoluciona y que resulta de la interacción entre una persona con una deficiencia y sus factores personales y contextuales, resultando en una restricción en su participación en igualdad de condiciones [11]. La participación es un derecho humano e incluye estar involucrado en todos los aspectos de la vida como lo son lo civil, económico, politico, social y cultural [4, 11]. Por su parte, la rehabilitation (artículo 26, Convención) es una estrategia que facilita la participación y está enfocada a mejorar la salud, restaurar y mantener el funcionamiento a largo plazo [4, 12, 13]. Un enfoque de rehabilitación integral incluye entonces la articulación entre servicios y programas en salud, empleo, educación y el sector social [4]. El Plan de Acción Global por la Discapacidad recnoce la falta de investigaciones que evidencien las necesidades reales de las PCD como una barrera para la implementación de servicios de rehabilitación efectivos [14]. En Latinoamérica, las investigaciones en rehabilitación son escasas [15] resultando en poco evidencia disponible que apoye las decisiones de política. Por consiguiente, se crean políticas públicas y programas que pueden no satisfacer las necesidades reales de la [3]. Específicamente en Colombia, la investigación en discapacidad está apenas iniciando, presenta muy baja traslación del conocimiento [16] y muy baja participación de las PCD [14]. En este sentido, existe una necesidad de generar evidencia de calidad y apropiada para el context para informar el diseño e implementación de políticas y programas en Latinoamérica [16].

Colombia ha tenido avances significativo con el desarrollo de su marco legal hacia la garantía de los derechos humanos de las PCD en las últimas décadas. Específicamente, la constitución de 1991, la firma (2009) y ratificación (2011) de la Convención que resultaron en la Ley Estatutaria de Discapacidad (2013) que llevó a la construcción de la Política Pública Nacional de Discapacidad [17] [18]. Sin embargo, la implementación de estos planeas está en etapas tempranas. El progreso reportado ante la ONU después de la ratificación de la Convención se centró principalmente en los cambios en el marco legal [19-21]. Los avances reportados en cuanto a la implementación de programas como por ejemplo educación inclusive, correspondían a casos individuales de éxitos en ciudades principales [19-21]. Tanto el gobierno como la sociedad civil reconocieron que persisten retos importantes para lograr un cambio sistémico hacia la inclusión y el enfoque de derechos [19-21]. La ONU, como parte de la respuesta, urgió al Estado colombiano a garantizar la accesibilidad universal y a hacer énfasis en el ámbito territorial [22]. Es por esto que es necesario identificar brechas locales para poder incidir en las acciones que se requieren [19].

En Colombia persiste la falta de datos confiables y actualizados sobre la discapacidad en el país [19, 20]. Con base en las estimaciones de la Organización Mundial de la Salud, la discapacidad afectaría al menos al 15% de la población del país (7.5 millones de colombianos) [3]. El censo poblacional del 2005 reporta una prevalencia 6,3% [23]; mientras que el registro para la caracterización y localización de las personas con discapacidad (registro voluntario) reporta a junio de 2018 un 2.8% [24]. Este registro recoge además información sobre las condiciones de vida, reportando que el 50% de los adultos con discapacidad presentan una discapacidad asociada a una condición física o de movilidad [24]. El 22% reportan que necesitan rehabilitación (definida como terapias o medicinas) y el 59% de ellos no asisten a ningún programa [24]. Solo el 12% trabajan, 2% culminaron un programa universitario y más del 80% perciben un salario menor del mínimo o no perciben ningun ingreso [24]. Estas cifras reflejan las profundas inequidades que viven las PCD a pesar del marco legal con enfoque de [24]. Moreno-Angarita et al, previo a la Ley Estatutaria de Discapacidad, exploraron el concepto y la práctica de rehabilitación entre actores como administradores de programas, PCD y académicos [25]. Este estudio resalta las discrepancias en la comprensión del concepto de rehabilitación entre los actores – solo de Bogotá, la capital del país - con un enfoque sobresaliente en el modelo médico de la discapacidad [25]. Hay poco conocimiento acerca de los retos de las PCD fuera de la capital del país. Con el fin de lograr la equidad, es imperative identificar brechas y desarrollar evidencia en las regions del país.

Alienados con las propuestas de la Agenda Colombiana de Investigación en Discapacidad [26], con este trabajo esperamos generar evidencia desde las experiencias relacionadas con el acceso a la rehabilitación en una región de Colombia que no ha sido explorada previamente y con la segunda prevalencia en discapacidad del país [24]. Nuestro enfoque está informado en evidencia previa en cuanto a los amplios retos vividos por adultos con discapacidad física. Con este lente, examinamos las percepciones únicas en cuanto al acceso a servicios de rehabilitación integral y la participación de las PCD. También exploramos las perspectivas en cuanto al acceso a la rehabilitación y la participación de las PCD de los cuidadores de PCD física, profesionales de rehabilitación y otros actores. El objetivo era identificar brechas en la implementación de programas de rehabilitación y las barreras para acceder a estos recursos que pudieran impactar la participación de las PCD.

**Métodos**

Se llevó acabo un estudio cualitativo para explorar las experiencias relacionadas con el acceso y el uso de servicios de rehabilitación integral y su impacto en la participación de las PCD en Envigado, Colombia. Este trabajo se desarrolló en colaboración con la organización comunitaria ALFIME. ALFIME ofrece terapia física, activdad física, psicología, asesoramiento legal, un programa de vida independiente y sesiones educativas para usuarios y sus familias. Estos programas son financiados con recursos públicos del municipio de Envigado y son ofrecidos a los usuarios a un costo de acuerdo a su capacidad de pago. Muchos usuarios reciben servicios sin costo. Es por esto que ALFIME es uno de los recursos principales para las personas con discapacidad del municipio independientemente de su estatus socioeconómico.

Muestra

Este estudio se ejecutó en el municipio de Envigado que cuenta con 240 mil habitantes y está ubicado en la zona metropolitana de Medellín (la segunda ciudad más grande del país). Envigado tiene un nivel de desarrollo menor al de Bogotá y Medellín [27] y el 40,5% de las PWD del municipio están excluidos del capital humano – definido como con deprivación de los derechos a la educación y la salud [28]. Por medio de un muestreo intencional identificamos participantes claves que representan diversos grupos en cuanto a género, edad y otras características. Administramos una encuesta sociodemográfica básica (Tabla 1) para capturar estas características e informar el muestreo. En Colombia, el estatus socioeconomic tiene 6 niveles (1-6). Para este estudio, se agruparon en nivel bajo (1 y 2), medio (3 y 4) y alto (5 y 6). Las PCD eran elegibles de participar si tenían entrer 18-44 años. Seleccionamos este grupo de edad porque en representan en el país el Segundo grupo de edad con mayor prevalencia de discapacidad despues de los adultos mayores. Además, presentan unas tasas muy bajas de acceso a la educación y al empleo a pesar de estar en sus años más productivos [24]. Por otro lado, la discapacidad física para este estudio se definió como una deficiencia permenente físico o de movilidad que afecta el cuerpo, los miembros superiors o inferiors, la destreza o la coordinación [24]. Similarmente, realizamos un muestreo intencional de cuidadores, profesionales de rehabilitación y otros actores que reflejaban diferentes experiencias brindando cuidados, servicios u otras iniciativas de interés para las PCD. Para este estudio, los profesionales de rehabilitación fueron definidos como aquellos involucrados en servicios y programas dirigidos a mejorar el funcionamiento de las PCD. La OMS reconoce la rehabilitación como intersectorial y que puede ser llevada acabo por profesionales de la salud en conjunto con profesionales de la educación, empleo, trabajo social y trabajadores de la comunidad en contextos de recursos limitados [29].

Tabla 1. Variables sociodemográficas incluidas en la encuesta por tipo de participante.

| Personas con discapacidad | Cuidadores | Profesionales de Rehabilitación | Otros actores |
| --- | --- | --- | --- |
| Edad  Género  Condición de salud  Productos de apoyo que utiliza  Estado civil  Estrato socioeconomico (bajo, medio, alto)  Nivel de educación  Recibe pension por discapacidad  Ocupación | Edad  Género  Horas brindando cuidado  Nivel de educación del cuidador  Estrato socioeconomico  Ocupación del cuidador | Edad  Género  Años trabajando con PCD  Área de ocupación (ejm. Salud, deporte, social) | Edad  Género  Tipo de organización donde trabaja (ONG, gobierno, academia, privada, consultor) |

Procedimientos

Las PCD y los cuidadores fueron remitidos por el personal de ALFIME e invitados a participar en el estudio. Los profesionales de rehabilitación representaron varias disciplinas y perspectivas, fueron invitados a participar del grupo total de profesionales afiliados con ALFIME. Otros actores fueron identificados por medio de las agencias del gobierno municipal, organizaciones de la sociedad civil organizada, la academia y líderes comunitarios.

Las entrevistas semi-estructuradas se llevaron a cabo en persona en español por uno de los miembros del equipo investigador [MLTH]. Desarrollamos una guía para la entrevista, con preguntas abiertas no directivas, para cada uno de los grupos de participantes. Las guías exploraban las áreas de independencia y autonomía, percepciones sobre la discapacidad, percepciones y disponibilidad de recursos de rehabilitación y ciudadanía. Miembros del equipo [MLTH, LVT, WCC] desarrollaron las guías con percepciones de expertos en ALFIME [remitirse al Additional file 1]. Los procedimientos descritos en este trabajo son parte de un proyecto macro que involucró activamente a la comunidad en un ejercicio de mapeo comunitario y un foro comunitario. Las entrevistas se realizaron en un espacio privado en ALFIME o en una ubicación de preferencia del entrevistado. Éstas duraron un máximo de 2 horas y 27 minutos, fueron grabadas digitalmente y luego transcritas literalmente. Notas de campo fueron diligenciadas inmediatamente después de cada entrevista. Los datos fueron administrados y analizados Dedoose Versión 8.0.35, aplicación web (2018). Antes de cada entrevista, cada participante diligenció una breve encuesta con preguntas sociodemográficas (Table 1).

Utilizamos análisis temático de contenido para analizar la información. Específicamente las técnicas de resúmenes analíticos, codificación abierto, identificación de códigos temáticos y desarrollo de libro de códigos. Cada entrevista fue codificada por dos miembros del equipo, las descrepancias fueron discutidas y resueltas por un tercer miembro. El análisis inicial nos permitió identificar los retos principales de acceso y uso de servicios de rehabilitación. Esto fue seguido por el análisis refinado de los temas enfocado en los dominios del significado de la rehabilitación, los retos de acceso a servicios y la participación comunitaria. Generamos matrices para comparar y contrastar las perspectivas de los participantes (PCD, cudidadores, profesionales de rehabilitación y otros actores). Evaluamos la saturación de los datos por medio de un proceso analítico iterativo que incluyó las entrevistas, revisar las notas de campo, la lectura y codificación de lainformación y el desarrollo de las matrices. El tamaño de nuestra muestra estuvo restringido por los recursos financiores; sin embargo, la evaluación de la saturación y la triangulación de los datos nos dan la confianza en que los temas claves lograron la saturación.

Los aspectos que definen la confiabilidad de los resultados están basados en los criterios propuestos por Nowel et. al [30]. Primero, MLTH asistió a ALFIME periódicamente lo que resultó en un compromiso prolongado, observación persistente y la construcción de una buena relación con la comunidad. Al menos un estudiante de pregrado asistió a las entrevistas y a la sesión de resumen inmediatamente luego de la misma junto con MLTH. Encuentros semanales de actualización se hiceron entre MLTH, MAM, and WCC. El procedimiento del contenido temático fue rigorozo, como se describió anteriormente. Todos estos aspectos establece la credibilidad. Segundo, la descripción completa del contexto y los métodos del estudio establecen la transferabilidad del mismo. Tercero, el equipo investigador auditó constantemente el process (ejm. Resúmenes analíticos, grabaciones de los datos crudos, notas de campo y transcripciones) y se llevó un diario con reflexiones. De esta manera se establede la fiabilidad. Por último, al lograr la credibilidad, transferabilidad y fiabilidad, se establece la confirmabilidad del estudio [30].

**Resultados**

Al estudio fueron invitados 35 participantes, tres hombres en el grupo de PCD no fueron elegibles por su edad o porque no contaban con los medios para asistir a la entrevista. Realizamos entrevistas con 8 participantes en cada grupo de interés, para un total de 32 participantes. La Tabla 2 presenta las características sociodemográficas de los 4 grupos. Para los participantes en el grupo de PCD, la condiciones de salud asociadas a la discapacidad inluyeron lesion medular, condiciones neurodegenerativas, artrítis reumatoide, entre otras. Todas las PCD utilizaban al menos una tecnología de apoyo (silla de ruedas manual, sillas de ruedas motorizada, caminador, etc) y la mitad eran solteros. Cuatro cuidadores proveian cuidado entre 1-3 horas al días, dos entre 4 y 6 houras al día, y dos más de 7 horas al día. Los profesionales de rehabilitación en promedio tenían 17 años de experiencia en el campo de la discapacidad; 4 proveían apoyo legal, individual o familiar y 4 estaban involucrados con educación, deporte o administración de programas. En el grupo de los otros actores, 2 estaban en la academia, 3 en el gobierno municipal y el resto eran consultores independientes.

Table 2. Características sociodemográficas de los participantes.

| Características/Participante | | PCD | Cuidadores | Profesionales de rehab* | Otros actores* |
| --- | --- | --- | --- | --- | --- |
| Edad (mediana (RIQ)) | | 32(5,5) | 51(12,5) | 52(4,5) | 36(5) |
| Género | Mujer | 6 | 6 | 7 | 5 |
|  | Hombre | 2 | 2 | 1 | 3 |
| Nivel educativo | Complete high school or less | 2 | 4 | Información no recolectada | |
|  | Associates degree or more | 6 | 4 |  |  |
| Nivel socioeco | Low | 2 | 1 | Información no recolectada | |
|  | Middle | 5 | 6 |  |  |
|  | High | 1 | 1 |  |  |
| Ocupación | | 2 estudiante  4 desempleado o sin ocupación  2 jubilado/pensionado | 4 cuidador/cuidado del hogar  2 desempleado o sin ocupación  1 jubilado/pensionado  1 empleado | 8 empleado | 8 empleado |

*RIQ: rango intercuartil; *Dos profesionales de rehabilitación y dos en los otros actores son personas con discapacidad.

Tres temas principales emergieron de las entrevistas: el significado de la rehabilitación, retos para acceder a los servicios y participación comunitaria. La Tabla 3 presenta el resumen de los temas por tipo de participante.

*Significado de la rehabilitación*

La rehabilitación fue conceptualizada de manera diferente en los grupos. Para las PCD, la rehabilitación está centrada en restaurar la función e incluye terapia física, practicar un deporte y acceder a la educación. La rehabilitación era vista como necesaria para supercar los miedos de salir de casa y de mejorar su propia vida. El valor de la rehabilitación se demuestra através de la interacción con pares que han sido exitosos con la rehabilitación física (pueden hacer las cosas mejora). Similarmente, para los cuidadores, la rehabilitación se relaciona con recobrar la función y está centrada en terapia física, psicológica y tecnologías de apoyo que ayudan a mejorar las habilidad físicas de una PCD. Por medio de la rehabilitación llega la aceptación de la discapacidad. Más importante, la rehabilitación significa menos trabajo para los cuidadores ya que la PCD puede hacer más tareas sin ayuda. Por otro lado, algunos cuidadores reconoces el efecto negative que puede tener hacer cosas en exceso por la PCD, interfieriendo con el proceso de rehabilitación.

Para los profesionales, la rehabilitación es una estrategia que promueve la independencia tanto de la PCD como de sus cuidadores y que debe ser diseñada a las necesidades individuales. Los profesionales ven la independencia no solo como la habilidad de realizar tareas sin ayuda, sino la capacidad de la PCD de tomar decisiones sobre su propia vida. Más allá, la rehabilitación es vista como un proceso multi-nivel e intersectorial en donde profesionales, familias y la comunidad trabajan mano a mano para lograr progreso. Desde su perspectiva la rehabilitación debe incluir terapia física, tecnología de apoyo, educación, empleo, deporte, arte y recreación, que además involucre los cuidadores y las familias. En el grupo de los otros actores tuvieron perspectivas similares a los profesionales de rehailitación. Los otros actores ven la rehabilitación – en la mayoría de los casos – como un proceso multi-nivel e intersectorial que “libera” a la PCD y su familia. Sin embargo, algunos profesionales y otros actores todavía perciben la rehabilitación como una estrategia para que la persona vuelva al punto previo de la lesion. Remítase al Additional file 2 para las citas textuales de este tema.

*Retos para acceder a servicios de rehabilitación integral*

Tres subtemas emergieron dentro de los retos para acceder a los servicios: barreras para la movilidad personas, percepciones y conocimiento sobre discapacidad y navegar el sistema. Referirse al archivo adicional 3 para leer ejemplos de testimonies que ilustran cada uno de los subtemas.

Barreras para la movilidad personal

Todos los participates estuvieron de acuerdo en los retos relacionados con la movilidad personal. Primero, las PCD viven en hogares inaccesibles: en pisos elevados sin ascensor o deben navegar escaleras para entrar/salir de casa. Estas barreras limitan significativamente la independencia en casa y para salir de casa. Segundo, la falta de transporte público accesible y asequible representa un reto constant para acceder a los servicios de rehabilitación disponibles y para participar en la comunidad. Las PCD, los profesionales de rehabilitación y los otros actores resaltaron los avances en el sistema de transporte público local: el metro cuenta con plataformas salvaescaleras y hay algunos buses con plataforma para personas con movilidad reducida. Sin embargo, también resaltaron que dichas plataformas no siempre funcionan, no todos los buses las tienen y la frecuencia de circulación de los buses accesibles es desconocida. Como consecuencia, las personas deben utilizar taxi (más costoso) y en algunos casos los taxistas no llevan la silla de ruedas. No tener los medios para costearse el transporte fue reportado como uno de los principales causantes de no asister a servicios de rehabilitación ni de participar en cominidad.

Además, todos los participantes reportaron que los espacios públicos (escuelas, universidades, restaurantes, salas de cine, estadios deportivos, etc) no eran accesibles ya que muchos tienen escaleras sin rampas o elevadores, las rampas eras muy inclinadas o los baños muy pequeños. Varios participantes en todos los grupos mencionaron que los andenes son inaccesibles, forzando a las personas a navegar en la ruta con los carros e incrementando el riesgo de un accidente. Se hizo mención también a las condiciones montañosas del municipio como una barrera. La combinación de los retos presentados tienen como resultado que la PCD requiera ayuda para poder salir de casa, incrementando los costos de participar en la comunidad al tener que incluir el tiempo del asistente y del transporte de ambos.

Algunas PCD, profesionales de rehabilitación y otros actores discutieron el impacto negative que tiene la falta de acceso a la tecnología de apoyo apropiada (prótesis y sillas de ruedas) que satisfagan las necesidades individuales de las personas y que reciban el entrenamiento apropiado en su uso.

Percepción y conocimiento sobre discapacidad

Las percepciones y el conocimiento sobre discapacidad emergieron como un reto para acceder a recursos por parte de todos los participantes. Las PCD reclaman la necesidad de ser reconocidos como individuos, sin etiquetas o clasificaciones derivadas de su condición de salud. Según las PCD y los cuidadores, algunos profesionales de la rehabilitación asumen que las personas con una misma condición de salud tienen el mismo funcionamiento, ignorando la voz de la PCD sobre sus necesidades y capacidades individuales. Varios cuidadores se refirieron al día en que su familiar fue dado de alta del hospital (luego de adquirir la discapacidad) y al hecho de no haber recibido suficiente capacitación para afrontar la nueva vida. Existe una desconfianza general hacia los médicos, debido a que los diagnósticos iniciales fueron dados con una connotación negativa y los desenlaces de sus vidas han sido mucho más de lo que predijeron los médicos. Los profesionales de rehabilitación perciben experiencias similares y describen como la falta de capacitación para trabajar con PCD resulta en una comunicación pobre que limita la información que es compartida por los profesionales con las PCD y sus familias. Desde el punto de vista de los profesionales de rehabilitación, es común que las PCD no reciban información sobre las posibilidades más allá del diagnóstico. Se hace un llamado de atención a que la formación actual en rehabilitación no presta atención suficiente a la sexualidad y actividades lúdicas.

Algunos participantes perciben que otros profesionales más allá del sector salud, como arquitectos e ingenieros, carecen de conocimiento sobre discapacidad y accesibilidad limitando el diseño universal de los caminos, edificios, casas o otros espacios físicos. Los profesionales de rehabilitación y los otros actores declaran que muchos programas públicos en educación, deporte y tecnologías de apoyo son administrados por personas sin experiencia trabajando con PCD. Capacitaciones en el tema de discapacidad y accesibilidad son una necesidad sentida. Los profesionales de rehabilitación y los otros actores reconocen que la responsabilidad de promover la participación no es solo de los profesionales y reclaman que muchas PCD y sus cuidadores no están interesados en aprender, no se esfuerzan lo suficiente y esperan que el gobierno y la sociedad les provean todo. Desde su perspectiva entonces esto lleva a limitaciones en la participación y a una desconexión entre las expectativas de las PCD y lo que los programas pueden ofrecer. Por ejemplo: varias PCD estaban esperando que profesionales del centro de empleo público les encontrarán un trabajo, pero no estaban buscando uno activamente. Otros actores resaltaron que las PCD requieren capacitación en cuanto a sus derechos y como indicir para que les sean garantizados y en auto-conomiento para poder comprender sus posibilidades individuales.

Navegar el sistema

Todos reconocieron que la ruta de atención para las PCD y sus familias no es clara. Los servicios de rehabilitaicón que son prescritos pueden tartarse varios meses en ser aprobados por el seguro médico y el número de sesiones que son aprobadas son percibidas como insuficientes. Impactando por ende la continuidad en los servicios. Sin embargo, hubo una contradicción en un punto de vista explicado por una PCD quien criticó la falta de objetivos claros en los procesos de rehabilitación. Sugieriendo que hay PCD que se quedan por años en el mismo programa de rehabilitación sin un objetivo claro por el cual trabajar. La mayoría de las PCD y los cuidadores mencionaron haber tenido que utilizar recursos legales (la tutela en Colombia) porque les fueron negados serivicios de rehabilitación y en algunos casos sin explicación. Otros actores estuvieron de acuerdo con que es recurrente tener que apelar legalmente porque la cobertura a los servicios prescritos son negados por el seguro médico. Los cuidadores por su parte compartieron además que algunos servicios no están incluidos por las aseguradoras como la equinoterapia y hay que pagar por ellas de manera particular. Una cuidadora manifesto su preocupación en el momento de la entrevista por un proyecto de ley que buscaba excluir la cobertura de las tecnologías de apoyo por parte del sistema de salud.

Por último, los profesionales de rehabilitación manifestaron que la discapacidad no es una prioridad en las agendas de política pública. Lo que afecta la continuidad de los programas que existen y los hace dependientes del político de turno. Se identifica además que hay una carencia de recursos para financiar el deporte (incluyendo el equipamento) y programas artísticos y de recreación. Los otros actores indicaron la falta de articulación entre programas y recursos.

*Participación en la comunidad*

Las PCD ven la participación como fundamental en su proceso de rehabilitación. De acuerdo con sus experiencias, la educación y el empleo son importantes porque los involucran con actividades sociales. Los otros actores mencionaron la importancia del empleo para mejorar la calidad de vida de las PCD y el entorno familiar, además del rol clave que tiene la educación en el mejoramiento de la participación. Sin embargo, algunas PCD y otros actores mencionaron que muchas compañíasprefieren no contratar PCD debido a la legislación que los protege de ser despedidos debido a su discapacidad. Remítase al Additional file 4 para ver ejemploe de citas que exploran la participación en la comunidad.

Varias PCD indicaron que las actividades que más disfrutan de participar en su comunidad están relacionadas con el ocio y la recreación (deporte, arte, compartir con amigos y familia). Los cuidadores estuvieron en acuerdo con este tipo de actividades. Las PCD, los profesionales de rehabilitación y los otros actores reconocieron la importancia del deporte para mejorar la independencia y crear un sentido de responsabilidad y compromiso. Los profesionales de rehabilitación y los otros actores recalcaron que la falta de interés de las PCD y sus cuidadores limita su propia participación en comunidad.

Las PCD creen que pueden ser influyentes al participar en actividades de toma de conciencia sobre discapacidad y al incidir para que sus comunidades sean más accesibles. Otro punto importante puesto sobre la mesa por las PCD, los profesionales de rehabilitación y los otros actores es la necesidad de quelas PCD lideren las iniciativas de discapacidad. Una persona en el group de PCD fue muy crítica reclamando que la mayoría de los programas de rehabilitación no contratan personas con discapacidad.

**Discusión**

Nuestros hallazgos evidencian los retos que perpetúan las inequidades en el acceso a los servicios de rehabilitación en una comunidad de PCD física en Colombia. A pesar de los avances significativos en el marco legal del país, continúan existiendo desconexiones importantes entre las necesidades de las PCD y las perspectivas de los demás actores. Por ejemplo, un decreto del 2003 obliga a que todos los buses de transporte público nuevos a partir del 2005 sean accesibles [31]. Nuestros resultados indican que algunas mejoras se han logrado, pero persiste la falta de transporte público accesible y consistente. La comprensión y la conciencia sobre la discapacidad por parte del sector púbico, la falta de compromiso político en el ámbito local y recursos límitados puede ser factores que contribuyan a la lenta implementación de las políticas públicas, sobre todo en los lugares fuera de las ciudades capitales.

La falta de consenso entre las PCD y los demás actores sobre el significado de la rehabilitación limita la efectividad de los efuerzos de incidencia para lograr el acceso equitativo a estos servicios. Puede ser además una indicación de que muchos todavía comprenden a la discapacidad desde el modelo médico: la persona con discapacidad debe ser reparada [32]. Un trabajo previo realizado en Bogotá antes de la Ley Estatutaria de Discapacidad también reportó una falta de consenso en el concepto del significado de rehabilitación entre líderes con discapacidad, hacedores de política pública, cuidadores, profesionales de educación inclusiva, académicos y otros profesionales [25]. Este estudio también reportó que la creencia general era que la rehabilitación dependía únicamente del sector salud [25]. Estas concepciones y conocimientos sobre discapacidad desactualizados que observamos en nuestros participantes son una indicación de que la cultura continua aferrada al modelo médico de la discapacidad [32], contribuyendo a las inequidades en el acceso a la rehabilitación que resulta en restricciones en la participación. Un reciente ejemplo es el proceso para obtener el certificado oficial de discapacidad de una persona en Colombia. A pesar de que la el marco conceptual de la CIF tiene casi 20 años, la primera regulación en el país que certifica la discapacidad basado en dicho marco fue en el 2018 [33]. Hay evidencia que soporta el hecho de que cuando los profesionales no son concientes de las capacidades y los derechos de las personas, se convierten en un obstáculo [20] debido a que no remiten a las personas a los servicios que requieren [34]. Este desconocimiento (barreras actitudinales) por parte de los profesionales ha sido igualmente reportado en zonas rules de Sudáfrica, Uganda y en otras regiones en Colombia [35-37].

Una potencial estrategia que promueva el cambio de paradigma sería incorporar explícitamente el tema de discapacidad en los currículos formales, de tal manera que los profesionales aprendan a trabajar con las PCD y sean facilitadores y no obstáculos para la participación de los mismos [38, 39]. Las instituciones de educación superior tienen la responsabilidad de asegurar que los nuevos profesionales – no solo los del área de la salud – sean concientes de las políticas locales y las tendencias globales con enfoque de derechos de las PCD [19, 25, 40]. Mayores esfuerzos se requieren para la toma de conciencia efectiva sobre la discapacidad como parte de la diversidad humana y como un asunto de derechos humanos, de acuerdo al mandato del artículo 4 de la Convención [4]. Es necesario un llamado a la acción donde se desarrollen colaboraciones público-privadas que promuevan la toma de conciencia sobre discapacidad y rehabilitación integral, con el enfoque de derechos [8]. En síntesis, el entendimiento de manera persistente de la discapacidad desde el modelo médico de la misma representa un riesgo al factor de aceptabilidad requerido para el acceso con equidad a la rehabilitación [6].

Es de anotar que a pesar de que la mayoría de los participantes definieron la rehabilitación desde una perspectiva del modelo médico de discapacidad, cuando se refieron a los retos para acceder a los servicios, dichos retos eran ubicados fuera del individuo (modelo social de la discpacidad) [41]. Específicamente, se resaltan factores contextuales críticos como la inaccesibilidad tanto dentro como fuera de casa (incluyendo el transporte público), la falta de tecnologías de apoyo apropiadas, creencias culturales y conocimiento general sobre la discapacidad y la falta de una ruta clara de atención [11]. Estos factores contextuales han sido reconocidos por las Naciones Unidas como retos para lograr vidas saludables para todos y todas [8]. Ejemplos en África identifican falta de transporte, falta de servicios, equipamento y medicamentos inadecuados y altos costos como barreras para las PCD acceder a la salud [42-44]. En Brasil la inaccesibilidad física en los hospitales ha sido reportada por PCD como una barrera que debe ser derrumbada [45]. En Tunja, Colombia, el uso de taxi por PCD fue 2.6 veces mayor que de las personas sin discapacidad debido a la inaccesibilidad de los buses públicos [46]. Chile recientemente hizo un llamado a la mejora del transporte público, incluyendo el metro, en su accesibillidad para las PCD [47]. Lograr la accesibilidad universal es un requisite para mejorar la equidad en el acceso a la salud sino también un prerequisite para la participación de las PCD. Esto se debe a que en contextos difíciles se requiere planeación intensive para ir a algún lugar debido a la inaccesibilidad del entorno construido [48]. Adicionalmente, los factores contextuales descritos anteriormente contribuyen a aumentar el costo de la participación y del acceso a la salud debido a la necesidad de requerir un asistente personal y transporte privado para llegar a un servicio [4, 35, 49-51]. Garantizar la movilidad personal de la manera más independiente posibles es un derecho humano en la Convención (artículo 20) [4] y un prerequisito para lograr los Objetivos de Desarrollo Sostenible [8, 52, 53].

La falta de una ruta de atención en rehabilitación formal para las PCD y sus familias impacta negativamente su habillidad de acceder oportunamente a los servicios de rehabilitación que requieren. La Ley Estatutaria de Discapacidad y la Política Nacional de Discapacidad responsabilizan al Ministerio de Salud y Protección Social a desarrollar un modelo de rehabilitación integral con directrices claras para que los diferentes sectores se articulen [17, 18]. A la fecha de publicación de este artículo, dicho modelo no había sido publicado. La falta de coordinación en la provision de los servicios afecta la disponibilidad de los recursos requeridos para el acceso a la rehabilitación [6, 8]. Se requiere el involucramiento de las PCD y los demás actores para el desarrollo e implementación del modelo que satisfaga las necesidades de las PCD. El Sistema Nacional de Discapacidad debe priorizar el desarro de este modelo, incluyendo una estrategia clara de monitoreo y evaluación [54].

La calidad de vida se ve impactada positivamente cuando uno participa en las actividades que disfruta [48]. Encontramos un desacuerdo entre las perspectivas de las PCD y las de los profesionales y de los otros actores con respecto al grado de participación, el liderazgo y la capacidad de autogestión de las PCD y sus familias. Los proveedores aseguran que todavía se requiere más compromiso e involucramiento de las PCD para participar, mientras que las PCD resaltan la existencia de barreras para llegar y participar en posiciones de liderazgo, incluso en programas para PCD. Los cuales deberían estar liderados por PCD de acuerdo con el movimiendo de vida independiente “nada sobre nosotros, sin nosotros” [55]. Las altas tasas de desempleo de las PCD en el país soportan el clamor de este grupo [24]. Es necesario promover de manera deliberada a aquellos individuos con discapacidad calificados para posiciones de liderazgo. El reciente decreto de cuotas de empleados con discapacidad en el sector público del país tiene el potencial de afectar este aspecto positivamente en el corto plazo [56].

La muestra permitió lograr la saturación de los temas de interés principal. Es por esto que logramos caracterizar la diversidad de perspectivas en cuanto a la rehabilitación integral de los actores que representan diferentes sectores de la comunidad. Además, logramos identificar brechas proponsas a implementar acciones. Debemos reconocer que el muestreo intencional pudo conyevar a seleccionar PCD, cuidadores, profesionales de la rehabilitación y otros actores que estaban más involucrados con la comunidad. Sin embargo, con la estrategia de muestreo - encontrar personas heterogéneas en género, nivel socioeconómico, discapacidad física, nivel de educación y rol - buscamos asegurar que los participantes representaran una diversidad de experiencias. La mayoría de los participantes fueron mujeres, lo cual es consistente con las diferencias de género en las profesiones sociales y de salud [57, 58] y en los roles de [59-61]. Sin embargo, es necesario anotar que en el nivel población hay más hombres que mujeres con discapacidad física en el rango de edad explorado en este estudio [24]. Otras investigaciones han reportado que las mujeres participan más en investigación que los hombres [62, 63], también puede sugerir que se forjó una mejor relación con las mujeres. La distribución del nivel socioeconómico de la muestra es similar a la reportada por las PCD en el país, 80% viven en hogares de nivel socioeconómico bajo [24].

Reconociendo que este estudio representa las experiencias del municipio de Envigado, los retos pueden ser transferibles a otros contextos que cuenten con un marco legal en discapacidad que esté evolucionando. Una de las limitaciones de nuestro trabajo es que las perspectivas presentadas solo son de adultos con discapacidad física. Con el fin de informar estrategias inclusivas dirigidas a reducir las inequidades en rehabilitación y en salud para todas las PCD, es necesario incluir las perspectivas de personas con otras deficiencias. Por ejemplo, para las personas sordas la comunicación puede ser una de las barreras más significativas para acceder equitativamente a la salud [64], mientras que para personas con discapacidad visual o intelectual, la información escrita (prescripción, planes caseros, remisiones, etc) pueden ser barreras importantes [8, 65]. Por último, las experiencias de los miembros de las fuerzas militares – hay un número significativo de miembros con discapacidad debido al conflict armado – podrían ser exploradas ya que el sistema de salud que los cobija es diferente al de la sociedad civil [66]. Por último, hubo temas que emergieron y que no alcanzaron la saturación como la sexualidad y que son relevantes para el acceso equitativo a la salud.

**Conclusiones**

La participación en comunidad es el fin último de la rehabilitación integral. Brechas significativas en la implementación del marco nacional de discapacidad tales como constructos desactualizados sobre discapacidad, retos persistentes en el entorno construido y la movilidad, falta de personal capacitado y dificultadas para comprender y navegar el sistema, continuan limitando el acceso a la rehabilitación integral. Al identificar estas brechas con potencial de generar acción, la comunidad puede empoderarse y liderar procesos y esfuerzos de incidencia para lograr el acceso con equidad a la rehabilitación integral por parte de las personas con discapacidad y sus familias.

**Referencias**

1. Comisión Económica para América Latina y el Caribe, *Informe regional sobre la medición de la discapacidad. Una mirada a los procedimientos de medición de la discapacidad en América Latina y el Caribe.* 2014: Santiago de Chile. p. 48.

2. World Health Organization, *World report on ageing and health.* Geneva: World Health Organization, 2015.

3. World Health Organization and World Bank, *World report on disability*, in *Geneva: WHO*. 2011.

4. United Nations, *Convention on the rights of persons with disabilities and optional protocol*. 2006, United Nations: New York, NY.

5. Comisión Económica para América Latina y el Caribe, *Panorama social de América Latina 2016*. 2017. p. 284.

6. Gulliford, M., et al., *What does' access to health care'mean?* Journal of health services research & policy, 2002. **7**(3): p. 186-188.

7. Mji, G., et al., *Exploring the interaction of activity limitations with context, systems, community and personal factors in accessing public health care services: A presentation of South African case studies.* African journal of primary health care & family medicine, 2017. **9**(1): p. 1-9.

8. United Nations, *Rights of persons with disabilities: Note by the Secretary-General*, in *Seventy-third session*, G. Assembly, Editor. 2018.

9. Eide, A.H. and S.H. Braathen, *Disability and equity in global health.* Tidsskrift for Den norske legeforening, 2017.

10. MacLachlan, M., H. Mannan, and E. McAuliffe, *Access to health care of persons with disabilities as an indicator of equity in health systems.* Open medicine, 2011. **5**(1): p. e10.

11. World Health Organization, *International classification of functioning, disability and health (ICF)*. 2001, Geneva: World Health Organization.

12. Stucki, G., et al., *Rehabilitation: The health strategy of the 21st century.* Journal of rehabilitation medicine, 2017.

13. World Health Organization, *Global disability action plan 2014-2021: Better health for all people with disability*. 2014: World Health Organization.

14. Organización Mundial de la Salud, *Discapacidad: Proyecto de acción mundial de la OMS sobre discapacidad 2014-2021: Mejor salud para todas las personas con discapacidad: Informe de la Secretaría*, Organización Mundial de la Salud, Editor. 2014.

15. Organización Panamericana de la Salud, *Discapacidad: lo que todos debemos saber.* Publicación Científica y Técnicas No. 616. 2006, Washington, D.C.: Organización Panamericana de la Salud,.

16. Velandia, I.C., et al., *Caracterización de investigaciones en discapacidad en Colombia 2005-2012.* Revista de la Facultad de Medicina, 2013. **61**(2): p. 101.

17. Consejo Nacional de Política Económica y Social República de Colombia and Departamento Nacional de Planeación, *CONPES 166 de 2013 Discapacidad e Inclusión Social*, Consejo Nacional de Política Económica y Social República de Colombia and Departamento Nacional de Planeación, Editors. 2013: Bogotá.

18. Gobierno Nacional, *Ley Estatutaria de Discapacidad 1618*, in *Bogotá*, Congreso de Colombia, Editor. 2013.

19. Correa-Montoya, L. and M.C. Castro-Martínez, *Discapacidad e inclusión social en Colombia: Informe Alternativo de la Fundación Saldarriaga Concha al Comité de Naciones Unidas sobre los derechos de las personas con discapacidad*. 2016, Fundación Saldarriaga Concha: Bogotá. p. 160.

20. Coalición Colombiana por la Implementación de la Convención sobre los Derechos de las Personas con Discapacidad, *Informe alternativo de la Coalición Colombiana para la implementación de la Convención sobre los Derechos de las Personas con Discapacidad*. 2016.

21. República de Colombia, *Informe inicial sobre la implementación de la Convención sobre los Derechos de las Personas con Discapacidad en Colombia*. 2013, Ministerio de Salud y Protección Social: Bogotá.

22. Cabeza-Monroy, G., et al., *Una mirada crítica del informe de Colombia al Comité sobre los Derechos de las Personas con Discapacidad.* Revista Española de Discapacidad (REDIS), 2017. **5**(2): p. 221-226.

23. DANE, *Censo General 2005.* Libro Censo General, 2005: p. 245-275.

24. Ministerio de Salud y Protección Social, *Sala situacional de las personas con discapacidad*. 2018: Bogotá. p. 37.

25. Moreno-Angarita, M., et al., *Análisis cualitativo del concepto y praxis de rehabilitación integral percibido por distintos actores involucrados.* Revista de la Facultad de Medicina, 2016. **64**: p. S79-84.

26. Cruz-Velandia, I., et al., *Hacia la formulación de una Agenda de Investigación en Discapacidad para Colombia.* Revista de la Facultad Nacional de Salud Pública, 2017. **35**(2).

27. Departamento Nacional de Planeación, *Tipologías Departamentales y Municipales: Una propuesta para comprender las entidades territoriales colombianas*. 2015, Dirección de Desarrollo Territorial Sostenible: Bogotá.

28. Beltrán, J.C.G. and C.I.G.d. B, *Discapacidad en Colombia: Reto para la Inclusión en Capital Humano. Tomo I*. 2008, Fundación Saldarriaga Concha: Bogotá.

29. World Health Organization, *WHO Guidelines on health-related rehabilitation (Rehabilitation Guidelines)*. 2011.

30. Nowell, L.S., et al., *Thematic Analysis: Striving to Meet the Trustworthiness Criteria.* International Journal of Qualitative Methods, 2017. **16**(1): p. 1609406917733847.

31. Ministerio de Transporte, *Decreto 1660 de 2003 Por el cual se reglamenta la accesibilidad a los modos de transporte de la población en general y en especial de las personas con discapacidad.* 2003, Ministerio de Transporte: Bogotá.

32. Albrecht, G.L., K.D. Seelman, and M. Bury, *Handbook of disability studies*. 2001: Sage Publications.

33. Ministerio de Salud y Protección Social, *Resolución 583 de 2018: Por la cual se implementa la certificación de discapacidad y el Registro de Localización y Caracterización de Personas con Discapacidad*. 2018.

34. Departamento Nacional de Planeación and Econometría Consultores, *Evaluación institucional de la política nacional de discapacidad complementada con una evaluación de resultados cualitativos sobre la población con discapacidad*. 2012: Bogotá. p. 215.

35. Mulumba, M., et al., *Perceptions and experiences of access to public healthcare by people with disabilities and older people in Uganda.* International Journal for Equity in Health, 2014. **13**(1): p. 76.

36. Grut, L., et al., *Accessing community health services: challenges faced by poor people with disabilities in a rural community in South Africa.* African Journal of Disability, 2012. **1**(1): p. 1-7.

37. Floyd, M.H., et al., *Identificación de las barreras del entorno que afectan la inclusión social de las personas con discapacidad motriz de miembros inferiores.* Salud Uninorte, 2012. **28**(2).

38. Morera, M.R., *Facilitadores (as) personales en el ámbito de la discapacidad, una experiencia cercana.* Reflexiones, 2012. **91**(2): p. 8.

39. Alfaro-Rojas, L., *Psicología y discapacidad: un encuentro desde el paradigma social.* Revista Costarricense de Psicología, 2013. **32**(1): p. 63-74.

40. Hammell, K.W., *Quality of life, participation and occupational rights: A capabilities perspective.* Australian occupational therapy journal, 2015. **62**(2): p. 78-85.

41. Hammel, J., et al., *Evaluating use and outcomes of mobility technology: a multiple stakeholder analysis.* Disabil Rehabil Assist Technol, 2013. **8**(4): p. 294-304.

42. Eide, A.H., et al., *Perceived barriers for accessing health services among individuals with disability in four African countries.* PLoS One, 2015. **10**(5): p. e0125915.

43. Visagie, S., et al., *Factors related to environmental barriers experienced by persons with and without disabilities in diverse African settings.* PloS one, 2017. **12**(10): p. e0186342.

44. Van Rooy, G., et al., *Perceived barriers to accessing health services among people with disabilities in rural northern Namibia.* Disability & Society, 2012. **27**(6): p. 761-775.

45. de Azevedo, T.R., et al., *Accesibilidad para personas con discapacidad física en los hospitales públicos.* Enfermería Global, 2015. **14**(37): p. 310-318.

46. D’otero, J.C.P., L.G.M. Díaz, and N.A.M. Peña, *Patrones de viaje y problemas de accesibilidad de personas en situación de discapacidad en Tunja.* Revista Lasallista de Investigación, 2017. **14**(2): p. 20-29.

47. Organización de Consumidores y Usuarios and Seguridad de Transporte y Minería, *Estudio de accesibilidad en transporte público para personas con movilidad reducida.* 2016: Rancagua. p. 47.

48. Jespersen, L.N., et al., *Living with a disability: a qualitative study of associations between social relations, social participation and quality of life.* Disability and rehabilitation, 2018: p. 1-12.

49. Hanass-Hancock, J., et al., *‘These are not luxuries, it is essential for access to life’: Disability related out-of-pocket costs as a driver of economic vulnerability in South Africa.* African journal of disability, 2017. **6**(1): p. 1-10.

50. Borg, J., S. Larsson, and P.O. Östergren, *The right to assistive technology: for whom, for what, and by whom?* Disability and Society, 2011. **26**(2): p. 151-167.

51. Rohwerder, B., *Assistive technologies in developing countries. K4D Helpdesk Report.* 2018, Institute of Development Studies: Brighton, UK.

52. Global Disability Rights Now. *Link between the Sustainable Development Goals and the Convention on the Rights of People with Disabilities*. 2016 [cited 2018 April 28]; Available from: <http://www.globaldisabilityrightsnow.org/infographics/link-between-sustainable-development-goals-and-crpd>.

53. United Nations. *Transforming our world: the 2030 agenda for sustainable development*. 2015 [cited 2015 September 26, 2015]; Available from: <https://sustainabledevelopment.un.org/post2015/transformingourworld>.

54. Congreso de Colombia, *Ley 1145 de 2007: Por medio de la cual se organiza el Sistema Nacional de Discapacidad y se dictan otras disposiciones.* 2007, Diario oficial 46,685: Bogotá.

55. Charlton, J.I., *Nothing about us without us: Disability oppression and empowerment*. 1998: Univ of California Press.

56. Ministerio de Trabajo, *Decreto 2011 de 2017: por el cual se reglamenta el porcentaje de vinculación laboral de personas con discapacidad en el sector público*. 2017: Bogotá.

57. Ministerio de Protección Social and Sociedad Médica para la Investigación y Control de Riesgos Profesionales y Ambientales, *Condiciones de salud y trabajo en el sistema general de riesgos profesionales.* 2007.

58. Arias, D., *Características de profesionales y auxiliares de enfermería del Huila. 2010.* Avances en Enfermería, 2012. **30**(2): p. 56-66.

59. Gómez-Galindo, A.M., O.L. Peñas-Felizzola, and E.I. Parra-Esquivel, *Caracterización y condiciones de los cuidadores de personas con discapacidad severa en Bogotá.* Revista de Salud Pública, 2016. **18**: p. 367-378.

60. Cardona-Arango, D., et al., *Características demográficas y sociales del cuidador en adultos mayores.* 2011.

61. Asociación Colombiana de Facultades de Fisioterapia ASCOFAFI and Asociación Colombiana de Fisioterapia ASCOFI, *Estudio nacional de condiciones de salud y trabajo de los fisioterapeutas*. 2015: Bogotá.

62. Randolph, S., T. Coakley, and J. Shears, *Recruiting and engaging African-American men in health research.* Nurse researcher, 2018. **25**(4).

63. Villa-Torres, L., P.J. Fleming, and C. Barrington, *Engaging men as promotores de salud: perceptions of community health workers among Latino men in North Carolina.* Journal of community health, 2015. **40**(1): p. 167-174.

64. London Assembly, *Access to health services for deaf people*, London Assembly, Editor. 2015: London. p. 34.

65. del Pilar Oviedo, M., M.L. Hernández, and M. Ruìz, *Baja visión en Colombia: una situación invisible para el país.* Facultad Nacional de Salud Pública, 2015. **33**(1).

66. Ministerio de Justicia, *Ley 1471 por medio de la cual se dictan normas relacionadas con la rehabilitación integral de los miembros de la Fuerza Pública, alumnos de las escuelas de formación de las Fuerzas Militares y sus equivalentes en la Policía Nacional, personal civil del Ministerio de Defensa Nacional y de las Fuerzas Militares y personal no uniformado de la Policía Nacional.*, Ministerio de Justicia, Editor. 2011: DIARIO OFICIAL. AÑO CXLVII. N. 48116. 30, JUNIO, 2011. PÁG. 112.
